# Long isoforms of the COPD risk gene FAM13A orchestrate human lung epithelial development

**DOI:** 10.1093/ajrcmb/aanag078

**Published:** 2026-05-04

**Authors:** Rhiannon B. Werder, Méline Homps-Legrand, Rebecca Hyatt, Jonathan Lindstrom-Vautrin, Carlos Villacorta-Martin, Pushpinder Bawa, Michael H. Cho, Xiaobo Zhou, Andrew A. Wilson

**Affiliations:** 1 Murdoch Children’s Research Institute, Melbourne, VIC 3052, Australia; 2 Center for Regenerative Medicine of Boston University and Boston Medical Center, Boston, MA 02118, USA; 3 The Pulmonary Center and Department of Medicine, Boston University School of Medicine, Boston, MA 02118, USA; 4 Channing Division of Network Medicine, Department of Medicine, Brigham and Women’s Hospital, Harvard Medical School

**Keywords:** FAM13A, lung epithelial development, induced pluripotent stem cells, Wnt/β-catenin

## Abstract

Impaired lung development and lung function can lead to the development of chronic obstructive pulmonary disease (COPD). Genome wide association studies (GWAS) have identified associations between variants in the gene *FAM13A* with both lung function and COPD. Of the major *FAM13A* isoforms expressed in humans, only the shorter isoforms are expressed in mice. This species difference has hindered investigations into whether full-length, human-specific isoforms contribute to human lung development, a question that remains unstudied to date. To functionally address this question, we disrupted the long isoform of *FAM13A* in human induced pluripotent stem cells (iPSCs). Specific loss of this isoform prevented the emergence in culture of mature airway or alveolar epithelial lineages in directed differentiation protocols. We demonstrate that the *FAM13A* long isoform is critical to patterning NKX2–1+ lung progenitor cells through dysregulating Wnt/β-catenin signaling during early stages of development *in vitro*. These findings provide the first evidence that the COPD risk gene *FAM13A* may be vital in the developing human lung epithelium.

## Introduction

Chronic obstructive pulmonary disease (COPD) is a leading cause of morbidity and mortality, with rates rising globally. The disease is characterized by fixed airflow obstruction due to progressive damage, inflammation and remodeling to the airways and alveoli. There are no therapies that prevent, arrest or reverse COPD progression, highlighting a clear need to better understand mechanisms underlying COPD pathogenesis. COPD susceptibility is the product of environmental exposures (e.g., tobacco smoke) and genetics (1–4). Increasingly, it is recognized that both genetic and environmental susceptibility in early life contribute to COPD onset in some individuals. This insight results from longitudinal cohort studies showing that a subset of individuals with COPD had impaired lung function in early adulthood (5) and that this impairment is associated with factors including low birth weight (6), childhood asthma and early life respiratory infections (7). Together, these findings suggest that COPD arises not only from accelerated lung function decline with age, compounded by environmental injury, but also from a failure to achieve peak lung function due to impaired lung development.

Genome wide association studies (GWAS) have consistently identified single nucleotide variants (SNVs) in *FAM13A* associated with low lung function, COPD and idiopathic pulmonary fibrosis (8–11). *FAM13A* expression increases during human lung development (12), although whether FAM13A actively contributes to this process is unknown. Unlike in mice, the human *FAM13A* locus includes 2 distinct transcriptional start sites contributing to expression of multiple *FAM13A* transcripts of varying length. Full length isoforms (hereto after referred to as “FAM13A-long”) contain a Rho GTPase activating protein (RhoGAP) domain (13), which is absent from “short” isoforms. In mice, only the short isoforms are present and *Fam13a* knockout mice undergo normal lung development (14). Increasing evidence suggests that the long isoforms of FAM13A found in humans have distinct functions (13), which remain to be explored in the context of lung development.

Studies to date have uncovered pleiotropic functions for FAM13A in the lung. FAM13A-long regulates airway cilia movement (13). FAM13A is linked to TGF-β mediated airway epithelial remodeling (15–17) and fatty acid oxidation (18). Several reports show that FAM13A modifies Wnt/β-catenin signaling albeit with seemingly discordant results. For instance, overexpression of FAM13A-short in Xenopus embryos or in immortal A549 cells stabilizes β-catenin (19) while deletion of *Fam13a* in mice leads to the accumulation and activation of β-catenin in alveolar epithelial progenitors (14, 20). These seemingly conflicting data may accurately represent divergent model systems or may reflect differences in FAM13A and β-catenin interactions in different cell types. Importantly, Wnt/β-catenin signaling controls emergence of lung progenitors in the mouse foregut (21, 22) and regulates specification of proximal and distal epithelial fates (23, 24) while inappropriate hyperactive Wnt drives non-lung lineages in the developing lung (25). As such, the potential contribution of FAM13A to regulation of β-catenin could have important consequences for human lung epithelial development and/or function.

Functional studies of human lung development are fraught with challenges, including access to tissue, ethical considerations, genetic tractability, and long-term expansion potential (26). To address these challenges, here we apply human induced pluripotent stem cells (iPSCs) to interrogate developmental mechanisms in lung epithelium. Directed differentiation of iPSCs to lung epithelial cells recapitulates key developmental milestones that occur *in utero*, passing through definitive endoderm and anterior foregut endoderm stages before specification of lung progenitors expressing the transcription factor, NKX2–1 (27–30). NKX2–1+ lung progenitors can then differentiate into mature epithelial lineages, including pseudostratified airway epithelial cells, type 1 and type 2 alveolar epithelial cells (31–34). To identify developmental contributions of FAM13A, here we apply CRISPR/Cas9 editing to iPSCs to knockout FAM13A-long, which is highly expressed in early development. We find that absence of FAM13A-long impairs differentiation of mature epithelial lineages due to aberrant patterning of NKX2–1+ lung progenitors driven by dysregulated Wnt/β-catenin signaling.

## Material and Methods

Detailed methods can be found in the supplement of this article.

### Human iPSC derivation and maintenance

All iPSCs exhibited a normal karyotype, confirmed via G-banding analysis (Cell Line Genetics). Detailed protocols for iPSC culture can be accessed at https://crem.bu.edu/cores-protocols/#protocols. The iPSCs used in this study are available upon request from the CReM iPSC repository at https://stemcellbank.bu.edu. All human iPSC work was approved by the Institutional Review Board of Boston University (protocol H33122).

### Gene editing

To create FAM13A-long mutants, BU3 NGST iPSCs were nucleofected (Lonza) with 5 ug of plasmid encoding CRISPR/Cas9 (Cas9–2A-GFP) and gRNA targeting exon 2 of *FAM13A* designed using CRISPOR software (v4.3). iPSCs were sorted for Cas9-GFP expression into 96 well plates coated with MEFs. Emergent clones were manually isolated, expanded and screened by PCR and Sanger Sequencing. To confirm indel zygosity, we cloned PCR products from iPSC clones of interest into a plasmid vector using the CloneJET PCR cloning kit (ThermoFisher) followed by Sanger Sequencing.

### Directed differentiation of human lung progenitors

To create NKX2–1+ lung progenitors, human iPSCs underwent directed differentiation to recapitulate developmental trajectories, as we have previously described (29, 32, 35, 36). By day 14–15 of the protocol, NKX2–1+ lung progenitors were specified. At this stage, cells were collected for RNA, fixed for immunostaining, or dissociated with 0.05% trypsin to sort for NKX2–1-GFP+ cells.

### Directed differentiation of human airway or alveolar epithelial cells

Following sorting, NKX2–1+ lung progenitors were resuspended in growth factor reduced Matrigel (Corning) to create droplets. After solidifying, droplets were covered with proximal airway or distal alveolar media (31–33). Cells were maintained in these media conditions for two weeks, with feeding every two days. Where indicated, cells were passaged using 0.05% trypsin, as described (35) then replated in Matrigel droplets in their respective medias.

### Single cell RNA sequencing

To multiplex samples, D6 cells were stained with hashing antibodies (Biolegend, #394631 and #394633). Sequencing of both libraries (GEX and HTO) was performed using an Illumina NextSeq 2000 instrument, pooled 50:1. Reads were processed with the Cellranger 3.0.2 pipeline and further analysis with Seurat (v 4.0.1). QC metrics are detailed in the Supplemental methods. We performed principal component analysis (PCA) on the sparse expression matrix and Uniform Manifold Approximation and Projection (UMAP) on the top 20 principal components. Differential gene expression was determined by a log fold-change of 0.25 with a Wilcoxon Rank Sum test and gene-set enrichment analysis (GSEA) was performed using the hypeR package (37). Regulon analysis was performed using the dorothea package (38). Data are deposited at Gene Expression Omnibus (GEO): GSE306210 (reviewer token: mbsfeicixpgfxgr).

### Statistics

All datasets were formally assessed for normality using Shapiro-Wilk tests and, where sample size permitted, Kolmogorov-Smirnov tests. One-way ANOVA followed by Tukey’s multiple comparisons test or Kruskal-Wallis test as appropriate was used for comparisons among three or more groups. Specific details can be found in each figure legend and in Supplemental Table 1. Statistical significance was defined as a p value of <0.05, with p values indicated on graphs as follows: *p < 0.05, **p < 0.01, ***p < 0.001. Data are presented as mean, with error bars representing the standard deviation (SD).

## Results

### Disruption of the FAM13A-long isoform in human iPSCs

**Figure 1:**
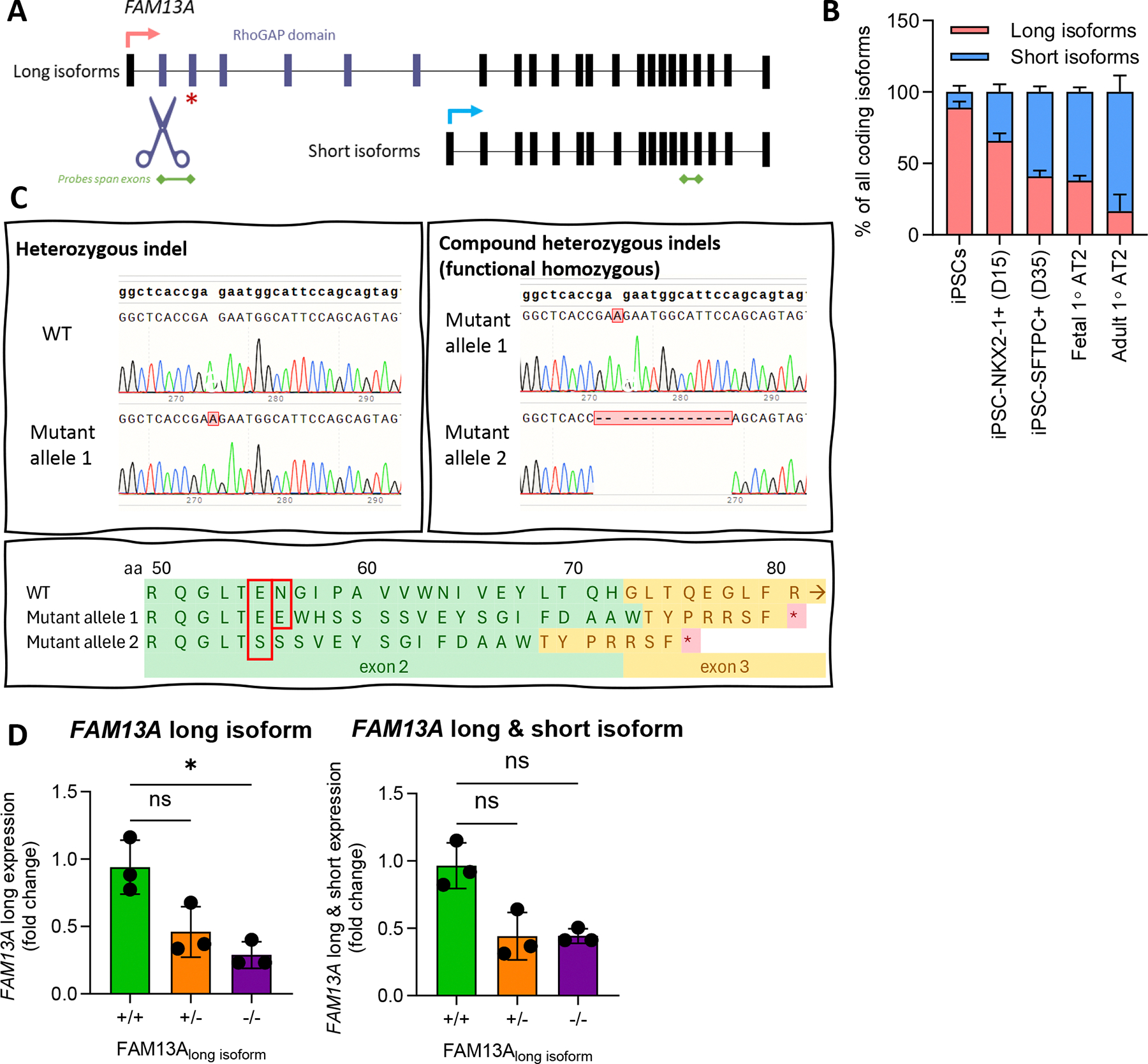
Generating induced pluripotent stem cells (iPSCs) deficient in the FAM13A long isoform. A) Isoforms of human *FAM13A* have separate transcriptional start sites. The long isoform contains a RhoGAP domain. CRISPR-Cas9 was used to create indels in exon 2 of the long isoform, introducing premature stop codons. Exons spanned by qRT-PCR primers denoted in green. B) Expression of long or short *FAM13A* isoforms in iPSCs, iPSC-derived lung progenitors (D15) or iPSC-derived type 2 alveolar epithelial cells (D35), or in primary fetal or adult type 2 alveolar epithelial cells (29, 32). C) Sanger sequencing traces of iPSCs with heterozygous and compound heterozygous indels in *FAM13A* and resulting changes to amino acids. D) Expression of long and short isoforms of *FAM13A,* quantified by qRT-PCR with the probes indicated in panel A, in iPSCs with heterozygous or compound heterozygous indels in FAM13A-long. n=3 experimental replicates; error bars represent SD. Statistical significance was determined by Kruskal-Wallis test; *p < 0.05.

To identify potential contributions of FAM13A-long to human lung development, we applied an *in vitro* differentiation system using human iPSCs. The human FAM13A gene has two annotated transcriptional start sites that result in expression of isoforms that vary in length (13, 39) (Figure 1A). To identify the expression patterns of these isoforms across human lung development, we analyzed published RNA sequencing datasets of iPSCs at 3 stages of differentiation together with fetal and adult human distal lung epithelium (Figure 1B and Supplemental Figure 1A) (29, 32). Interestingly, FAM13A-long expression was most abundant in iPSCs and iPSC-derived NKX2–1+ lung progenitors compared with later stages of the differentiation and in mature *in vivo* type 2 alveolar epithelial cells, where short isoform expression predominated (Figure 1B and Supplemental Figure 1A). This expression pattern suggested a role for FAM13A-long early in development. To disrupt expression of FAM13A-long in iPSCs, we applied CRISPR/Cas9 to introduce indels in exon 2 (Figure 1A). We isolated and expanded clones containing either heterozygous or compound heterozygous indels in FAM13A resulting in altered amino acid sequences and associated premature stop codons introduced in exon 3 (Figure 1C). These disruptions resulted in stepwise reductions of expression of FAM13A-long in heterozygous and compound heterozygous (hereafter referred to as knockout) mutant iPSCs, likely due to nonsense-mediated decay caused by newly-introduced premature stop codons (Figure 1D). Heterozygous and knockout mutant iPSCs were confirmed to be karyotypically normal (Supplemental Figure 1B), creating an isogenic set of iPSCs to investigate the function of FAM13A-long in lung development.

### Airway and alveolar epithelial cell specification are perturbed with FAM13A-long isoform insufficiency

**Figure 2:**
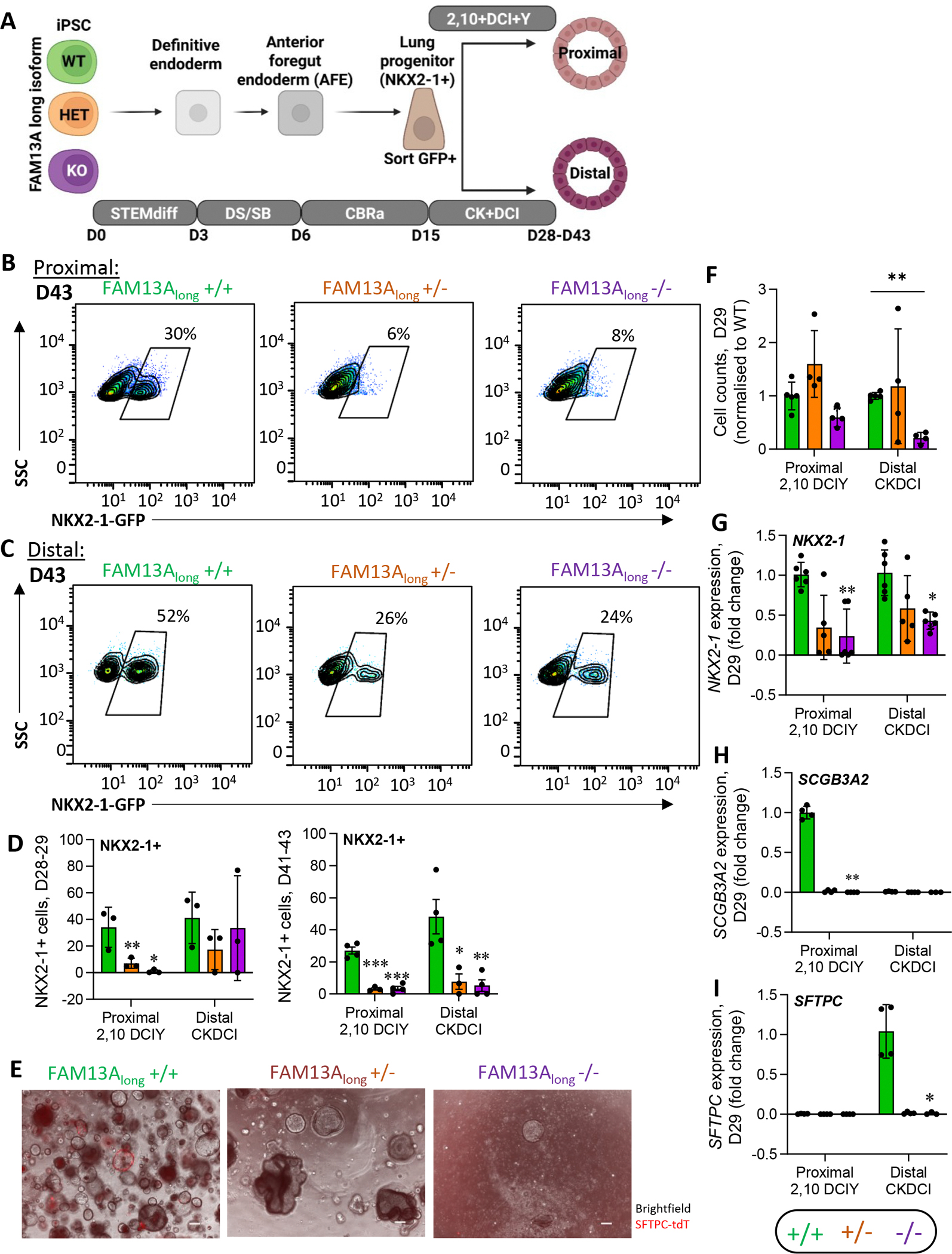
FAM13A-long regulates mature epithelial cell differentiation from iPSCs. A) Schematic of the directed differentiation of induced pluripotent stem cells (iPSCs) to proximal or distal lung epithelial cells. FAM13A-long wildtype (WT), heterozygous (HET) or knockout (KO) mutant iPSCs were differentiated to definitive endoderm then anterior foregut endoderm and then lung progenitors. NKX2–1+ lung progenitors were sorted and replated in proximal (2,10+DCI+Y) or distal (CK+DCI) medias for 2–4 weeks. B) Retention of NKX2–1-GFP+ cells after 4 weeks in proximal media, quantified by flow cytometry. C) Retention of NKX2–1-GFP+ cells after 4 weeks in distal media, quantified by flow cytometry. D) NKX2–1-GFP+ cells quantified by flow cytometry on D28–29 (top) or D41–43 (bottom). E) Live cell imaging of alveolospheres derived from NKX2–1+ lung progenitors after 4 weeks in distal media (D43). Scale bar = 200 μm. Brightfield (grey), SFTPC-tdTomato reporter (red). F) Total cell counts after 2 weeks in proximal or distal media, normalized to the WT condition in each media for each differentiation. G) Expression of *NKX2–1*, H) *SCGB3A2* or I) *SFTPC* following two weeks in proximal or distal media, quantified by qRT-PCR. n=3–6 independent differentiations; error bars represent SD. Statistical significance was determined by one-way ANOVA with a Tukey multiple comparison test or Kruskal-Wallis test after normality testing; *p < 0.05, **p < 0.005, ***p < 0.001.

Since proximal and distal epithelial fates are regulated by Wnt/β-catenin signaling (23, 24) and FAM13A can regulate this pathway (14, 19, 20), we first examined whether FAM13A-long altered lung epithelial differentiation capacity. iPSC-derived airway and alveolar epithelial cells can be derived *in vitro* via sequential exposure to growth factors identified in the developing embryo to pattern lung epithelium (29, 30, 32, 33, 40). These protocols require first the induction and sorting of lung progenitors expressing the transcription factor NKX2–1, followed by further maturation to either proximal airway or distal alveolar epithelial lineages (Figure 2A). To test the relative contribution of FAM13A-long to differing lung compartments, we isolated and cultured NKX2–1 expressing progenitors in either proximal or distal differentiation media. When differentiated in proximal airway epithelial media, iPSCs insufficient for FAM13A-long expression (heterozygous or full knockout) demonstrated a reduction in NKX2–1 retention (Figure 2B). We noted similar trends in NKX2–1 retention when heterozygous or knockout FAM13A-long iPSCs were grown in distal alveolar epithelial media (Figure 2C). This significant reduction in retention of NKX2–1+ cells among FAM13A-long mutants was observed across 4 independent differentiations (Figure 2D). FAM13A-long knockout cells failed to proliferate and/or survive in distal media conditions. This finding was confirmed quantitatively by cell counts and *MKI67* expression, which were lower in the KO cultures, and through live-cell imaging, which revealed markedly fewer alveolospheres in these conditions (Figure 2E–F and Supplemental Figure 2A). Moreover, we found that expression of mature genes associated with epithelial identity (e.g., *SCGB3A2* in airway cells, *SFTPC* in alveolar cells) were significantly reduced in cells lacking FAM13A-long following culture in proximalizing or distalizing media conditions (Figure 2G–I). Taken together, the defect in NKX2–1+ lung progenitors to give rise to mature lineages suggested that FAM13A-long may contribute to early lung epithelial patterning.

### Insufficiency of the FAM13A long isoform disrupts patterning of NKX2–1+ lung progenitors

We have previously shown that knockdown of FAM13A-long in mature iPSC-derived type 2 alveolar epithelial cells does not cause cells to lose epithelial identity (41). Therefore, we postulated that the failure of FAM13A-long deficient cells to generate mature airway or alveolar epithelial cells likely indicated a defect earlier during lung differentiation since FAM13A-long was absent from the start. We measured efficiency of endoderm induction in the early stages of differentiation and did not observe any differences between FAM13A-long sufficient or insufficient iPSCs (Supplemental Figure 2B). To ascertain whether FAM13A-long knockout iPSCs had inherent inabilities to differentiate to other lineages, we also tested the ability of these cells to differentiate to mesoderm, ectoderm, and another endoderm lineage (hepatocytes) (42, 43) and found that FAM13A-long insufficiency only significantly impacted mesoderm induction (Supplemental Figure 2C–E).

**Figure 3:**
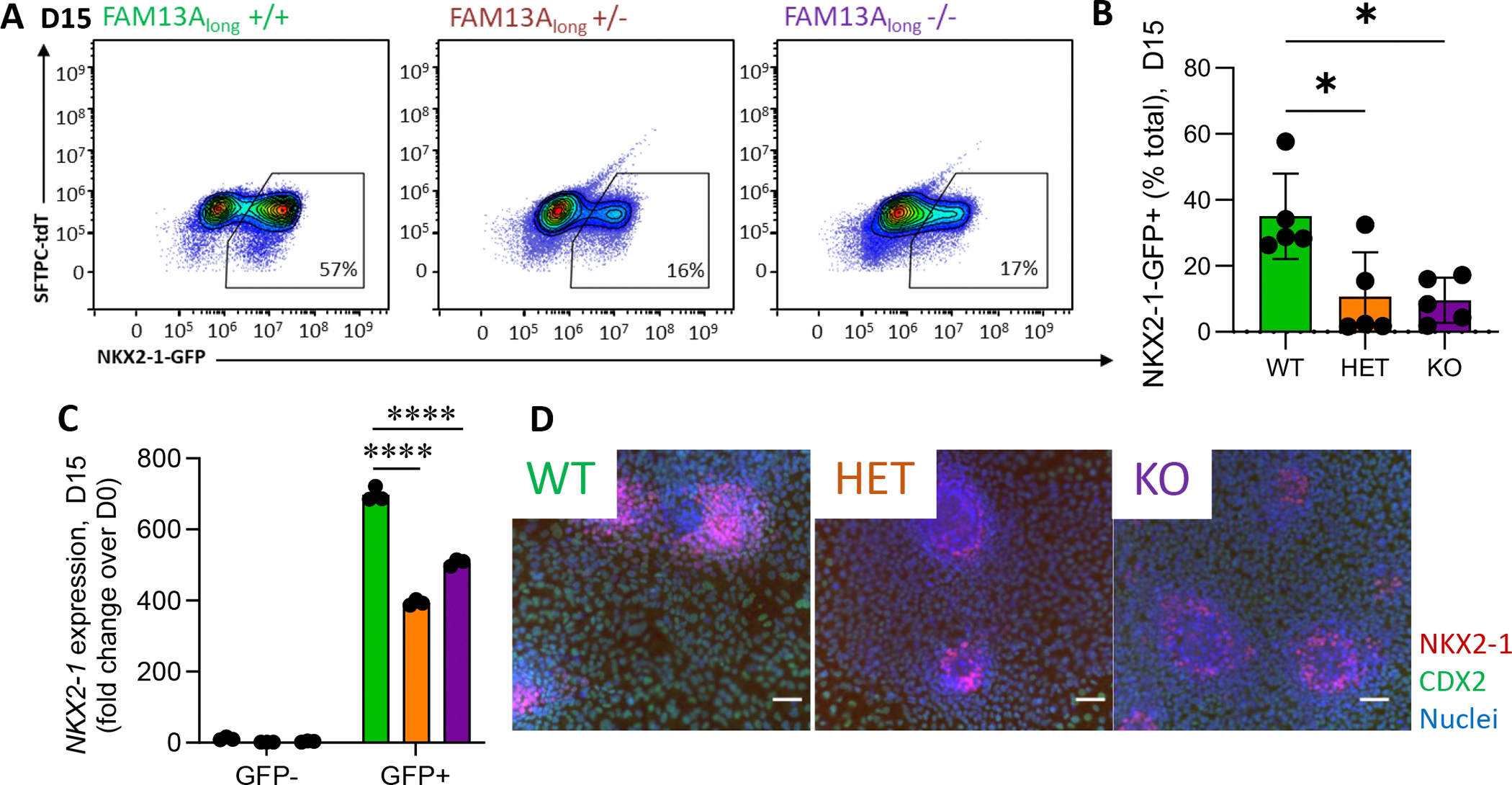
NKX2–1+ lung progenitors patterning is influenced by FAM13A-long. A) FAM13A-long isoform wildtype (WT), heterozygous (HET) or knockout (KO) mutant iPSCs were differentiated to lung progenitors. NKX2–1-GFP+ cells were detected by flow cytometry and B) quantified across 5 independent differentiations. C) NKX2–1+ (GFP+) and NKX2–1− (GFP−) cells were sorted (n=3 independent replicates) and expression of *NKX2–1* quantified in each fraction by qRT-PCR, relative to an average of D0 samples. D) Immunostaining for lung progenitors expressing NKX2–1+ in “islands” (red) surrounded by non-lung endoderm expressing CDX2 (green). Scale bar = 50 μm. Error bars represent SD. Statistical significance was determined by one-way ANOVA with a Tukey multiple comparison test; **p < 0.005, ****p < 0.0001.

During differentiation toward mature airway or alveolar epithelial lineages, we observed that FAM13A-long-deficient cells generated fewer NKX2–1+ lung progenitors at day 15 of the differentiation. Given that NKX2–1 is a master regulator of lung epithelial identity and is the earliest transcription factor expressed in the developing lung buds (44), we carefully quantified the efficiency of NKX2–1 induction during directed differentiation in FAM13A-long WT or knockout cells using a reporter iPSC line. Across multiple differentiations, we observed decreased numbers of NKX2–1+ lung progenitors with associated reduced expression of *NKX2–1* in sorted NKX2–1-GFP+ cells at day 15 of differentiation in FAM13A-long heterozygous or knockout cells (Figure 3A–C). Furthermore, we found that expression of the FAM13A-long isoform was significantly reduced in knockout cells in NKX2–1-GFP+ cells (Supplemental Figure 3A).

We next explored whether off-target lineages were arising in the lung differentiations at the expense of appropriate NKX2–1 induction for lung specification. Because NKX2–1 is also expressed in the developing thyroid and forebrain, we first investigated whether non-lung, NKX2–1+ cells might arise in the absence of FAM13A-long. In NKX2–1+ (GFP+) cells sorted on day 15, we did not find significantly increased expression of thyroid (*PAX8*) or brain (*OTX2*) markers in FAM13A-long insufficient cells (Supplemental Figure 3B–C). Interestingly, during differentiation of FAM13A-long insufficient iPSCs, the appearance of NKX2–1+ cells diverged from the usual dense clusters observed in the wildtype cells (Figure 3D). Non-lung endoderm lineages that typically arise in these cultures express CDX2 (45). While we observed CDX2+ cells adjacent to NKX2–1+ cells across all genotypes, the absolute number of cells expressing CDX2 was unaltered by the presence or absence of FAM13A-long (Figure 3D and Supplemental Figure 3D), suggesting alterations observed in NKX2–1+ cultured cells were not due to outgrowth of CDX2+ cells. We also probed the NKX2–1 negative cells for expression of canonical markers of other non-lung endoderm lineages including liver (*AFP*), intestine (*CDX2*), stomach (*TFF1*), esophagus (*TP63*) and pancreas (*PDX1*). However, we did not observe a significant increase in the expression of any of these markers in FAM13A-long insufficient cells at day 15 (Supplemental Figure 3E–I). Of note, *FOXA2* expression, a pan-endoderm marker, was unchanged between wildtype and knockout FAM13A-long cells (Supplemental Figure 3J). These findings confirm that FAM13A-long does not impact endoderm induction or maintenance yet plays a significant role in the specification of NKX2–1+ lung progenitors.

### Aberrant Wnt signalling resulting from FAM13A long isoform insufficiency impairs lung progenitor patterning

**Figure 4:**
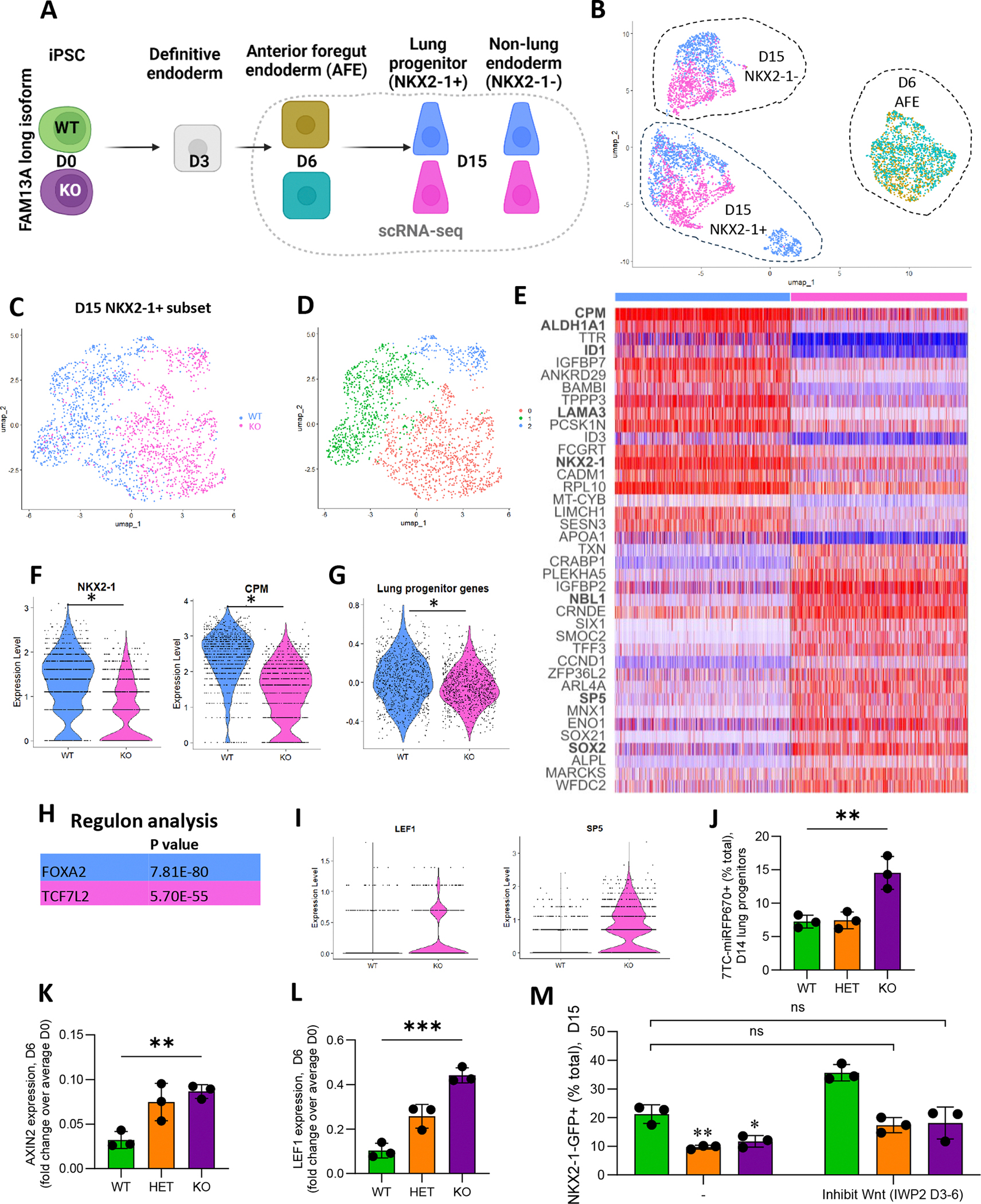
Loss of FAM13A-long leads to inappropriate Wnt/β-catenin signaling which disrupts normal lung development. A) FAM13A-long wildtype (WT) or knockout (KO) mutant iPSCs were differentiated to anterior foregut endoderm (D6, AFE) or lung progenitors (D15) prior to capture for 10X single-cell RNA-sequencing (scRNA-seq). B) Uniform manifold projection (UMAP) of D6 AFE cells, D15 NKX2–1− cells and D15 NKX2–1+ cells. C) UMAP of D15 NKX2–1+ cells that are FAM13A-long WT (blue) or KO (pink). D) Louvain clustering resolution of 0.2 of D15 NKX2–1+ cells. E) Heatmap showing top 20 differentially expressed genes between FAM13A-long WT (blue) and KO (pink) D15 NKX2–1+ cells. F) Normalized *NKX2–1* and *CPM* single cell expression in D15 NKX2–1+ cells. G) Module score of lung progenitor genes (29) in D15 NKX2–1+ cells. H) Top regulon of FAM13A-long WT (blue) or KO (pink) D15 NKX2–1+ cells. I) Normalized *LEF1* and *SP5* single cell expression in D15 NKX2–1+ cells. J) 7TC-miRFP670+ cells were quantified by flow cytometry in D14 NKX2–1+ cells. K) *AXIN2* and L) *LEF1* expression quantified by qRT-PCR in D6 AFE cells from FAM13A-long wildtype (WT), heterozygous (HET) or knockout (KO) mutant iPSCs. M) AFE cells were treated with IWP2 (from D3–6 of the differentiation), then NKX2–1-GFP+ lung progenitors were quantified on D15 by flow cytometry. n=3 experimental replicates; error bars represent SD. Statistical significance was determined by one-way ANOVA with a Tukey multiple comparison test; *p < 0.05, **p < 0.005, ***p < 0.001.

To explore molecular pathways modulated by FAM13A-long in lung epithelial progenitor development, we performed single-cell RNA sequencing (scRNA-seq). To maximize the contrast between FAM13A-long sufficiency and complete loss, FAM13A-long sufficient (WT) and knockout (KO) iPSCs were differentiated and collected at the anterior foregut endoderm (AFE) stage (day 6) or sorted at day 15 of lung epithelial differentiation to collect both NKX2–1+ and NKX2–1− cells (29) (Figure 4A and Supplemental Figure 4A). As expected, dimensionality reduction displayed cells clustering according to the stage of the differentiation (Figure 4B). While AFE cells appeared largely similar between genotypes, NKX2–1+ lung progenitors separated according to the presence or absence of FAM13A-long (Figure 4B). Indeed, when we reclustered only NKX2–1+ cells (Figure 4C), dimensionality reduction and Louvain clustering revealed two major clusters, separated by genotype (Figure 4C–D and Supplemental Figure 4B). More than 200 genes were differentially expressed between NKX2–1+ FAM13A-long WT and KO cells (Figure 4E). Canonical lung progenitor genes, *NKX2–1* and *CPM*, were among the top genes downregulated in FAM13A-long KO lung progenitors (Figure 4E–F), consistent with our earlier findings (Figure 3). Interestingly, *SOX2*, also expressed in developing human lung bud progenitors, (46, 47) was upregulated in KO lung progenitors. Nevertheless, overall expression of lung progenitor genes (29) was significantly reduced in FAM13A-long KO cells (Figure 4G and Supplemental Figure 4C). Gene-set enrichment analysis (GSEA) revealed that epithelial development programs were upregulated in WT relative to FAM13A-long KO lung progenitor cells (Supplemental Figure 4D).

We questioned whether FAM13A-long KO would impact the transcriptome of the non-lung endoderm cells that arise during the differentiation; therefore, we analysed the NKX2–1 negative cells collected at day 15 (Supplemental Figure 5A). Cells clustered according to cell cycle and genotype (Supplemental Figure 5B–C). Differentially expressed genes between the WT and KO cells included the upregulation of hepatic-associated genes in FAM13A-long sufficient cells (e.g., *AFP, APOB, APOA2*) or gut-associated genes in KO cells (e.g., *TFF3*) (Supplemental Figure 5D). However, as we had previously observed, FAM13A-long insufficient cells did not have an obvious upregulation of non-lung endoderm lineages (Supplemental Figure 5E–G), nor did they upregulate markers associated with iPSC-derived intestinal epithelium (45) (Supplemental Figure 5F). Taken together, these data confirm that the failure to pattern NKX2–1+ lung progenitors in the absence of FAM13A-long is not due to competition of non-lung endoderm lineages.

To understand the inappropriate patterning of lung epithelial progenitors in FAM13A-long KO cells, we conducted a regulon analysis to identify upregulated transcription factor (TF) networks based on expression of transcriptional targets. The top regulon in the WT NKX2–1+ cells was FOXA2, consistent with known interactions between the FOXA family of transcription factors with NKX2–1 (48), while the top regulon in FAM13A-long KO cells was TCF7L2, an effector of Wnt/β-catenin signaling (Figure 4H and Supplemental Figure 4E). Consistent with this finding, we found that Wnt/β-catenin target genes (e.g., *LEF1*, *SP5*) were significantly upregulated in FAM13A-long insufficient lung progenitors (Figure 4I). To functionally validate these observations, we transduced differentiating iPSCs with a 7TC reporter lentiviral construct to read out Wnt/β-catenin signaling (49) and observed a significant upregulation in activity in FAM13A-long KO lung progenitors (Figure 4J).

There is a delicate balance of spatial, temporal, and signaling strength dynamics of Wnt/β-catenin activity required for lung epithelial progenitor specification whereby an initial suppression of Wnt after endoderm induction permits foregut patterning but is then followed by Wnt activation to induce NKX2–1+ lung progenitors and drive distal fate decisions (21–25). Given this known sequence, we sought to understand whether earlier dysregulation of this pathway was occurring during the directed differentiation of cells lacking FAM13A-long. AFE cells at day 6 of differentiation appeared quite similar between genotypes (Supplemental Figure 6A–E), except that among the differentially expressed genes we observed upregulation of genes involved in Wnt activation (e.g., *WLS*) and Wnt targets (e.g., *SP5, CCDN1*) in FAM13A-long KO cells (Supplemental Figure 6B and F). Across multiple independent differentiations, we confirmed that the Wnt/β-catenin target genes, *AXIN2* and *LEF1*, were upregulated in AFE cells from FAM13A-long KO cells (Figure 4K–L). Finally, we sought to determine whether blocking Wnt/β-catenin signaling in FAM13A-long KO AFE cells would restore appropriate induction of NKX2–1+ lung progenitors. From the definitive endoderm stage to AFE stage, differentiations were treated with IWP2, a Porcupine inhibitor which blocks Wnt/β-catenin signaling (50). Strikingly, IWP2 treatment of heterozygous or FAM13A-long knockouts during this window restored the induction of NKX2–1+ cells at day 15 to wildtype levels (Figure 4M), suggesting that dysregulated Wnt/β-catenin signaling in the absence of the long isoform of FAM13A inappropriately patterns lung progenitors (50).

## Discussion

*FAM13A* SNVs are reproducibly associated by GWAS with lung function and COPD and impaired lung development often precedes COPD development (5, 8–11). While FAM13A expression is associated with human lung development (12), the role of the full-length isoform remains understudied. Using the directed differentiation of iPSCs to lung epithelial cells, we provide the first characterization of the FAM13A-long isoforms in human epithelial development. FAM13A-long regulates lung epithelial progenitor patterning and subsequent efficiency to generate mature epithelial lineages of the proximal and distal epithelium. Interestingly, this effect occurred through imbalanced Wnt/β-catenin activity starting in the developing anterior foregut endoderm cells. Our results *in vitro* offer the first indication that FAM13A could play a crucial role in the development of human lung epithelium.

Our findings suggest that FAM13A-long regulates canonical Wnt/β-catenin signaling in anterior foregut endoderm which is sustained into the lung progenitor phase. During gut tube patterning, high Wnt signaling promotes posterior patterning at the expense of anteriorization (51, 52), a finding that has been recapitulated in human pluripotent stem cell systems (27, 53). Although dysregulated Wnt/β-catenin was apparent at the anterior foregut endoderm stage of FAM13A-long mutants, the strength of the signal was likely not sufficient to induce posterior markers like *CDX2*. Instead, the dysregulated signaling appeared to disrupt subsequent patterning of lung progenitors. Future studies will be necessary to elucidate the mechanisms underlying this finding. Interestingly, we also observed a major defect in the capacity of FAM13A-long mutant iPSCs for mesodermal differentiation, which relies on Wnt signaling (54). Previous studies have shown that the short isoform of FAM13A directly interacts with protein phosphatase 2A (PP2A), to regulate Wnt/β-catenin signaling (14, 55). Whether similar mechanisms contributed to altered Wnt/β-catenin signaling observed in developing lung epithelial progenitors or in mesodermal lineages will be important to define in future experiments. Importantly, dysregulated Wnt/β-catenin signaling has been reported in COPD (56, 57), suggesting a potential mechanistic link between FAM13A-long function and altered epithelial patterning in disease.

In the absence of FAM13A-long we found that the quantity of NKX2–1+ lung progenitors was significantly reduced. Interestingly, the few NKX2–1+ cells that were derived also exhibited decreased *NKX2–1* transcript expression. Because of this, we were unable to disentangle whether NKX2–1 insufficiency was entirely responsible for the impaired differentiation of mature epithelial cells, or whether this defect also arose due to the need for appropriate Wnt signaling to pattern proximal and distal cells (33). NKX2–1 mutations in humans are associated with a spectrum of lung disease from surfactant homeostasis dysfunction to abnormal lung development and respiratory failure (58–61) and the complete absence of Nkx2–1 in mice results in hypoplastic lungs (62). These findings reinforce the concept that calibrated expression of NKX2–1 and its interactions with promoters of essential lung genes (e.g., surfactant genes) are integral to appropriate lung cell fate commitment and differentiation (60, 63). Thus, the reduction in *NKX2–1* expression in our *FAM13A* mutant cells suggests that FAM13A-long deficiency diminishes both the quantity and functional capacity of lung progenitors.

Different roles of FAM13A isoforms in lung epithelial cells are beginning to be recognized. Knockout of the short (and only) isoform in mice increases proliferation of alveolar epithelial progenitors (14, 20). In contrast, we have previously shown that knockdown of FAM13A-long in iPSC-derived type 2 alveolar epithelial cells slows their proliferation (41). Recently, FAM13A-long was found to coordinate cilia movement in the airway epithelium (13). Interestingly, the same authors found that FAM13A isoforms are differentially expressed in the human airway epithelium (13). Although scRNA-seq datasets have illuminated cell-type specific expression of *FAM13A*, including in individuals with COPD (20), the 3’-bias of these technologies has meant that FAM13A isoforms have been less explored. Furthermore, while the FAM13A COPD risk allele is associated with increased expression of FAM13A in the adult lung, no studies to date have investigated whether this SNV influences expression during lung development, nor if this SNV preferentially influences expression of FAM13A-long or -short isoforms. While our study could distinguish expression of the long isoform, we could not specifically measure the short isoform because our assay targeted a sequence shared by both isoforms. Given the varied roles that FAM13A isoforms appear to play in the development and function of the human lung epithelium, assays capable of distinguishing isoforms should be incorporated in future research.

Our studies to functionally determine the contribution of FAM13A-long to human lung development leveraged the capabilities of iPSCs and would have been challenging to conduct in human cells via existing alternative experimental platforms. The directed differentiation of iPSCs recapitulates developmental milestones of the developing lung (29, 32, 33, 64) and have been previously applied to study the pathogenesis of congenital lung diseases (32, 65, 66). To our knowledge, this is the first investigation using iPSCs to study the contribution of a COPD GWAS gene to epithelial development. Since genes associated with COPD by GWAS are commonly found to contribute to lung developmental pathways (66, 67), this approach could prove useful to study other genes in the future, especially when expression patterns of the gene of interest is specific to humans, limiting the application of mouse models (e.g., *HHIP* (67)). Moreover, future studies could apply patient-derived iPSCs with common FAM13A variants to assess their impact on lung epithelial development.

In conclusion, we report a previously unappreciated role for the FAM13A long isoform in human epithelial development. Through regulating the patterning and induction of NKX2–1 in lung progenitors via Wnt/β-catenin, FAM13A-long controls proximal and distal epithelial lineage specification. Our findings show the first functional association between a COPD GWAS gene with *in vitro* human lung development.

## Supplementary Material

Supplementary Table 1

Supplementary Methods

Supplementary Figures
