# Supplementary material for: Long isoforms of the COPD risk gene FAM13A orchestrate human lung epithelial development
Source: Am J Respir Cell Mol Biol. Author manuscript; Available in PMC 2026 Jul 6. (PMC13336431; doi:10.1093/ajrcmb/aanag078)
Supplement: Supplementary Methods [file NIHMS2185259-supplement-Supplementary_Methods.docx]

# **Material and Methods**

## *Human iPSC derivation and maintenance*

Human iPSCs were cultured on plates coated with growth-factor-reduced Matrigel (Corning) in mTeSR1 medium (StemCell Technologies). Routine cell passaging was performed using the Gentle Cell Dissociation Reagent (StemCell Technologies), and all iPSCs exhibited a normal karyotype, confirmed via G-banding analysis (Cell Line Genetics). Detailed protocols for iPSC culture can be accessed at https://crem.bu.edu/cores-protocols/#protocols. The iPSCs used in this study are available upon request from the CReM iPSC repository at https://stemcellbank.bu.edu. All human iPSC work was approved by the Institutional Review Board of Boston University (protocol H33122).

## *Gene editing*

To create heterozygous or homozygous knockouts of FAM13A-long, BU3 NGST iPSCs were nucleofected with 5 ug of plasmid encoding CRISPR/Cas9 (Cas9-2A-GFP) and gRNA targeting exon 2 of *FAM13A* (sequence: CGGCAGGGGCTCACCGAGAATGG) which were designed using CRISPOR software (v4.3). iPSCs in log phase growth were pretreated with 10 uM Y-27632 (Tocris) for 3 hours then dissociated to single cell with Gentle Cell dissociation reagent (StemCell Technologies). Cells were resuspended in DNA with P3 solution (Lonza) prior to nucleofection with 4-D nucleofector system using code CB-150 (Lonza). Cells were replated on plates coated with mitomycin C inactivated mouse embryonic fibroblasts (MEFs). Two days later, iPSCs were sorted for Cas9-GFP expression into 96 well plates coated with MEFs. Emergent clones were manually isolated, expanded and screened by PCR.

The following primers were used for PCR screening for indels (F = CGGCAGGGGCTCACCGAGAATGG; R = CGGCAGGGGCTCACCGAGAATGG) using Accuprime polymerase (ThermoFisher), prior to confirmation of the introduction of indels by Sanger Sequencing. To confirm whether indels were heterozygous or homozygous, we cloned PCR products from iPSC clones of interest into a plasmid vector using the CloneJET PCR cloning kit (ThermoFisher). For each iPSC clone, ten bacterial colonies were selected, plasmid DNA isolated using a Miniprep kit (QIAGEN) and Sanger Sequenced.

## *Directed differentiation of human lung progenitors*

To create NKX2-1+ lung progenitors, human iPSCs underwent directed differentiation to recapitulate developmental trajectories, as we have previously described (29, 32, 35, 36). In brief, iPSCs were differentiated to definitive endoderm over 3 days, marked by cKit+ CXCR4+, using the STEMdiff Definitive Endoderm Kit (StemCell Technologies). Cells were then passaged using Gentle Cell Reagent, and further cultured on growth factor reduced Matrigel (Corning)-coated plates. For three days, cells were in anteriorisation media containing 2 mM Dorsomorphin (Stemgent) and 10 mM SB431542 (Tocris) in complete serum-free differentiation medium (cSFDM) base media. In some experiments, this was supplemented with 5 uM IWP2 (Tocris). Cells were then cultured for an additional ten days in 3 mM CHIR99021 (Tocris), 10 ng/mL recombinant human BMP4 (R&D Systems), and 100 nM retinoic acid (Sigma) in cSFDM. By day 14-15 of the protocol, NKX2-1+ lung progenitors were specified. At this stage, cells were collected for RNA, fixed for immunostaining, or dissociated with 0.05% trypsin to sort for NKX2-1-GFP+ cells. Cells were resuspended in sort buffer (Hank’s Balanced Salt Solution [ThermoFisher], 2% FBS, 10 mm Y-27632, and 10 mM calcein blue AM [Life Technologies]) and then sorted using a MoFlo Astrios.

## *Directed differentiation of human airway or alveolar epithelial cells*

Following sorting, NKX2-1+ lung progenitors were resuspended in growth factor reduced Matrigel (Corning) to create droplets. After solidifying, droplets were covered with proximal airway or distal alveolar media. Proximal airway media consisted of 250 ng/mL FGF2 (R&D Systems), 100 ng/mL FGF10 (R&D Systems), 50 nM dexamethasone (Sigma), 0.1 mM 8-Bromoadenosine 30,50 cyclic monophosphate sodium salt (Sigma), 0.1 mM 3-Isobutyl-1methylxanthine (IBMX; Sigma) and 10 uM Y-27632 (Tocris) in cSFDM (31, 33). Distal alveolar media contained 3 mm CHIR99021, 10 ng/mL rhKGF (R&D Systems), 50 nM dexamethasone (Sigma), 0.1 mM 8-Bromoadenosine 30,50 cyclic monophosphate sodium salt (Sigma), and 0.1 mM IBMX (Sigma) in cSFDM (32). Cells were maintained in these media conditions for two weeks, with feeding every two days. Where indicated, cells were passaged using 0.05% trypsin, as described (35) then replated in Matrigel droplets in their respective medias.

## *Flow cytometry*

Staining was quantified using a Stratedigm S1000EXI or a MoFlo Astrios (Beckman Coulter) and analysed with FlowJo v10.6.2 (FlowJo, Tree Star Inc) or FlowLogic v8.7 (Inivai Technologies). Plots shown represent single cells based on forward-scatter/side-scatter gating.

## *Induction of mesoderm or ectoderm*

To determine the potential of FAM13A long isoform mutant cells to create mesoderm or ectoderm germ layers, we used the STEMdiff Trilineage Differentiation kit, as per manufacturer’s instructions. Ectoderm was assessed by PAX6+ Nestin+ and mesoderm was determined by Brachyury+ NCAM+ by flow cytometry.

## *Directed differentiation of hepatocytes*

iPSCs were differentiated to iHeps using previously established protocols (37, 38). A detailed protocol for the hepatocyte differentiation of iPSCs is available for free download at: http://www.bu.edu/dbin/stemcells/protocols.php. In brief, iPSCs were differentiated to definitive endoderm over four days using the STEMdiff Definitive Endoderm kit (StemCell Technologies). Cells were then replated in hepatocyte differentiation media using stage-specific growth factors for directed differentiation to hepatocytes (38). At the end of the differentiation, hepatocytes were dissociated and analysed on flow cytometry for intracellular expression of alpha fetoprotein (AFP) and FOXA1.

## *FAM13A isoform analysis*

To quantify the expression of *FAM13A* isoforms, we used the RSEM package to quantify isoform abundance (39). Since FAM13A-long utilises a different promoter >235 kilobasepairs 5’ upstream of the short isoform promoter, we analysed previously published RNA sequencing datasets with paired-end reads that included undifferentiated iPSCs, iPSC-derived lung progenitors, iPSC-derived type 2 alveolar epithelial cells, and primary fetal and adult type 2 alveolar epithelial cells (29, 32) (GEO accession: GSE83310, [GSE96642](https://www.ncbi.nlm.nih.gov/geo/query/acc.cgi?acc=GSE96642)).

## *Real-Time Quantitative Reverse Transcription PCR*

Samples were collected in Qiazol (Qiagen, 79306) and stored at -80°C before RNA extraction using the RNeasy Plus Mini Kit following the manufacturer’s instructions (Qiagen, 74104). Complementary DNA (cDNA) was synthesized using MultiScribe Reverse Transcriptase (Applied Biosystems, 4374967). Quantitative RT-qPCR was performed for 40 cycles using predesigned TaqMan probes (Applied Biosystems). Probes used in this study were: *SFTPC* (Hs00161628_m1), *NKX2-1* (Hs00968940_m1), *FAM13A* (Hs01596554_m1), *FAM13A* (Hs00208453_m1), *SCGB3A2* (Hs0036978_m1), *PAX8* (Hs00247586_m1), *OTX2* (Hs00222238_m1), *AFP* (Hs01040598_m1), *TFF1* (Hs00907239_m1), *CDX2* (Hs01078080_m1), *TP63* (HS00978340_m1), *PDX1* (Hs00426216_m1), *FOXA2* (Hs05036278_s1), *AXIN2* (Hs00610344_m1), *LEF1* (Hs01547250_m1). Average Ct values for technical triplicates were calculated and normalized to internal 18S control. Fold change was determined over control cells using 2^-ΔΔCt.

## *Immunostaining*

Cells were fixed with 4% PFA for 20 minutes at room temperature. Samples were washed with PBS, permeabilised with 0.3% Triton and blocked with 4% normal donkey serum (NDS). Primary antibodies were diluted in 4% NDS and incubated overnight at 4°C. Primary antibodies used were: NKX2-1 (Dako, #M3575 8G7G3/1) and CDX2 (Abcam, #ab76541 EPR2764Y). Sections were washed PBS then incubated with fluorescent-conjugated secondary antibodies for 1 hour at room temperature before counterstaining with Hoechst 33342. Cells were imaged with a Keyence BZ-X700 fluorescence microscope and images were processed in Image J and Fiji.

## *7TC lentiviral reporter*

To monitor Wnt/β-catenin activity we adapted a TOPFlash system (40, 41). In brief, 7xTcf binding sites followed by a minimal promoter and miRFP670 (42) were cloned into a third-generation lentiviral vector (43). To track transduced cells, a constitutive TagBFP was also introduced to the vector. Lentivirus was produced as previously described (44). Definitive endoderm cells were transduced on day 3 of the differentiation with MOI 20. NKX2-1-GFP+ TagBFP+ miRFP670+/- cells were analysed on day 14 of the lung differentiation by flow cytometry.

## *Single cell RNA sequencing*

FAM13A-long wildtype and homozygous knockout cells were differentiated to anterior foregut endoderm (day 6 of the lung progenitor differentiation protocol) or NKX2-1+ lung progenitors (day 15 of the lung progenitor differentiation protocol) for single-cell RNA-sequencing (scRNA-seq). Cells were dissociated with 0.05% trypsin (D15) or Accutase (D6). To multiplex samples, D6 cells were incubated with Fc block (Biolegend, #422301) and stained with hashing antibodies (Biolegend, #394631 and #394633). All cells were sorted to collect live cells based on Zombie NIR (Biolegend, #423106) on a MoFlo Astrios cell sorter (Beckman Coulter). Prior to capture, hashed cells were pooled 1:1. Libraries were prepared as per the 10x Genomics scRNA-Seq 3’v3.1 and HTO protocols and quantified by a Kapp kit. Sequencing of both libraries (GEX and HTO) was performed using an Illumina NextSeq 2000 instrument, pooled 50:1. The sequencing generated reads with 94%≥Q30, and an average of 40,575 mean reads per cell. To generate fastq files and count matrices (combining gene expression and antibody capture libraries for each sample) we used the Cellranger 3.0.2 pipeline. For further processing and downstream analyses, we used Seurat (v 4.0.1). The HTODemux function was used to demultiplex samples based on hashing antibody expression. We excluded cells if they were negative for a HTO, positive for more than one HTO (annotated as doublets), if more than 15% of UMIs mapped to mitochondrial genes, if fewer than 800 genes were detected and/or based on potential doublets (based on abnormally high number of genes detected in proportion to cell density, as per 10X Genomics recommendations). To normalize the data, we used the regularized negative binomial regression method (SCTransform function) and regressed out cell degradation (mitochondrial percentage). We performed principal component analysis (PCA) on the sparse expression matrix and Uniform Manifold Approximation and Projection (UMAP) on the top 20 principal components. Clustering was computed using the Louvain algorithm at different resolutions, ranging from 1.5 to 0.05. Differential gene expression was determined by a log fold-change of 0.25 with a Wilcoxon Rank Sum test and gene-set enrichment analysis (GSEA) was performed using the hypeR package (45). Regulon analysis was performed using the dorothea package (46). Data are deposited at Gene Expression Omnibus (GEO): GSE306210 (reviewer token: mbsfeicixpgfxgr).

## *Statistics*

All datasets were formally assessed for normality using Shapiro-Wilk tests and, where sample size permitted, Kolmogorov-Smirnov tests. One-way ANOVA followed by Tukey’s multiple comparisons test or Kruskal-Wallis test as appropriate was used for comparisons among three or more groups. Specific details can be found in each figure legend and in Supplemental Table 1. Statistical significance was defined as a p value of <0.05, with p values indicated on graphs as follows: *p < 0.05, **p < 0.01, ***p < 0.001. Data are presented as mean, with error bars representing the standard deviation (SD).

# **References**

1. Regan EA, Lynch DA, Curran-Everett D, Curtis JL, Austin JH, Grenier PA, Kauczor HU, Bailey WC, DeMeo DL, Casaburi RH, Friedman P, Van Beek EJ, Hokanson JE, Bowler RP, Beaty TH, Washko GR, Han MK, Kim V, Kim SS, Yagihashi K, Washington L, McEvoy CE, Tanner C, Mannino DM, Make BJ, Silverman EK, Crapo JD. Clinical and Radiologic Disease in Smokers With Normal Spirometry. *JAMA Intern Med* 2015; 175: 1539-1549.

2. Zhou JJ, Cho MH, Castaldi PJ, Hersh CP, Silverman EK, Laird NM. Heritability of chronic obstructive pulmonary disease and related phenotypes in smokers. *Am J Respir Crit Care Med* 2013; 188: 941-947.

3. Bakke PS, Baste V, Hanoa R, Gulsvik A. Prevalence of obstructive lung disease in a general population: relation to occupational title and exposure to some airborne agents. *Thorax* 1991; 46: 863-870.

4. Tan WC, Sin DD, Bourbeau J, Hernandez P, Chapman KR, Cowie R, FitzGerald JM, Marciniuk DD, Maltais F, Buist AS, Road J, Hogg JC, Kirby M, Coxson H, Hague C, Leipsic J, O'Donnell DE, Aaron SD. Characteristics of COPD in never-smokers and ever-smokers in the general population: results from the CanCOLD study. *Thorax* 2015; 70: 822-829.

5. Lange P, Celli B, Agusti A, Boje Jensen G, Divo M, Faner R, Guerra S, Marott JL, Martinez FD, Martinez-Camblor P, Meek P, Owen CA, Petersen H, Pinto-Plata V, Schnohr P, Sood A, Soriano JB, Tesfaigzi Y, Vestbo J. Lung-Function Trajectories Leading to Chronic Obstructive Pulmonary Disease. *N Engl J Med* 2015; 373: 111-122.

6. Barker DJ, Godfrey KM, Fall C, Osmond C, Winter PD, Shaheen SO. Relation of birth weight and childhood respiratory infection to adult lung function and death from chronic obstructive airways disease. *Bmj* 1991; 303: 671-675.

7. Svanes C, Sunyer J, Plana E, Dharmage S, Heinrich J, Jarvis D, de Marco R, Norbäck D, Raherison C, Villani S, Wjst M, Svanes K, Antó JM. Early life origins of chronic obstructive pulmonary disease. *Thorax* 2010; 65: 14-20.

8. Hobbs BD, de Jong K, Lamontagne M, Bosse Y, Shrine N, Artigas MS, Wain LV, Hall IP, Jackson VE, Wyss AB, London SJ, North KE, Franceschini N, Strachan DP, Beaty TH, Hokanson JE, Crapo JD, Castaldi PJ, Chase RP, Bartz TM, Heckbert SR, Psaty BM, Gharib SA, Zanen P, Lammers JW, Oudkerk M, Groen HJ, Locantore N, Tal-Singer R, Rennard SI, Vestbo J, Timens W, Pare PD, Latourelle JC, Dupuis J, O'Connor GT, Wilk JB, Kim WJ, Lee MK, Oh Y-M, Vonk JM, de Koning HJ, Leng S, Belinsky SA, Tesfaigzi Y, Manichaikul A, Wang X-Q, Rich SS, Barr RG, Sparrow D, Litonjua AA, Bakke P, Gulsvik A, Lahousse L, Brusselle GG, Stricker BH, Uitterlinden AG, Ampleford EJ, Bleecker ER, Woodruff PG, Meyers DA, Qiao D, Lomas DA, Yim J-J, Kim DK, Hawrylkiewicz I, Sliwinski P, Hardin M, Fingerlin TE, Schwartz DA, Postma DS, MacNee W, Tobin MD, Silverman EK, Boezen HM, Cho MH, Investigators CO, Investigators E, LifeLines I, Group SR, International CGNI, Investigators UKB, International CGC. Genetic loci associated with chronic obstructive pulmonary disease overlap with loci for lung function and pulmonary fibrosis. *Nat Genet* 2017; 49: 426-432.

9. Wain LV, Shrine N, Artigas MS, Erzurumluoglu AM, Noyvert B, Bossini-Castillo L, Obeidat M, Henry AP, Portelli MA, Hall RJ, Billington CK, Rimington TL, Fenech AG, John C, Blake T, Jackson VE, Allen RJ, Prins BP, Campbell A, Porteous DJ, Jarvelin MR, Wielscher M, James AL, Hui J, Wareham NJ, Zhao JH, Wilson JF, Joshi PK, Stubbe B, Rawal R, Schulz H, Imboden M, Probst-Hensch NM, Karrasch S, Gieger C, Deary IJ, Harris SE, Marten J, Rudan I, Enroth S, Gyllensten U, Kerr SM, Polasek O, Kahonen M, Surakka I, Vitart V, Hayward C, Lehtimaki T, Raitakari OT, Evans DM, Henderson AJ, Pennell CE, Wang CA, Sly PD, Wan ES, Busch R, Hobbs BD, Litonjua AA, Sparrow DW, Gulsvik A, Bakke PS, Crapo JD, Beaty TH, Hansel NN, Mathias RA, Ruczinski I, Barnes KC, Bosse Y, Joubert P, van den Berge M, Brandsma CA, Pare PD, Sin DD, Nickle DC, Hao K, Gottesman O, Dewey FE, Bruse SE, Carey DJ, Kirchner HL, Jonsson S, Thorleifsson G, Jonsdottir I, Gislason T, Stefansson K, Schurmann C, Nadkarni G, Bottinger EP, Loos RJ, Walters RG, Chen Z, Millwood IY, Vaucher J, Kurmi OP, Li L, Hansell AL, Brightling C, Zeggini E, Cho MH, Silverman EK, Sayers I, Trynka G, Morris AP, Strachan DP, Hall IP, Tobin MD. Genome-wide association analyses for lung function and chronic obstructive pulmonary disease identify new loci and potential druggable targets. *Nat Genet* 2017; 49: 416-425.

10. Cho MH, McDonald ML, Zhou X, Mattheisen M, Castaldi PJ, Hersh CP, Demeo DL, Sylvia JS, Ziniti J, Laird NM, Lange C, Litonjua AA, Sparrow D, Casaburi R, Barr RG, Regan EA, Make BJ, Hokanson JE, Lutz S, Dudenkov TM, Farzadegan H, Hetmanski JB, Tal-Singer R, Lomas DA, Bakke P, Gulsvik A, Crapo JD, Silverman EK, Beaty TH. Risk loci for chronic obstructive pulmonary disease: a genome-wide association study and meta-analysis. *Lancet Respir Med* 2014; 2: 214-225.

11. Soler Artigas M, Loth DW, Wain LV, Gharib SA, Obeidat M, Tang W, Zhai G, Zhao JH, Smith AV, Huffman JE, Albrecht E, Jackson CM, Evans DM, Cadby G, Fornage M, Manichaikul A, Lopez LM, Johnson T, Aldrich MC, Aspelund T, Barroso I, Campbell H, Cassano PA, Couper DJ, Eiriksdottir G, Franceschini N, Garcia M, Gieger C, Gislason GK, Grkovic I, Hammond CJ, Hancock DB, Harris TB, Ramasamy A, Heckbert SR, Heliovaara M, Homuth G, Hysi PG, James AL, Jankovic S, Joubert BR, Karrasch S, Klopp N, Koch B, Kritchevsky SB, Launer LJ, Liu Y, Loehr LR, Lohman K, Loos RJ, Lumley T, Al Balushi KA, Ang WQ, Barr RG, Beilby J, Blakey JD, Boban M, Boraska V, Brisman J, Britton JR, Brusselle GG, Cooper C, Curjuric I, Dahgam S, Deary IJ, Ebrahim S, Eijgelsheim M, Francks C, Gaysina D, Granell R, Gu X, Hankinson JL, Hardy R, Harris SE, Henderson J, Henry A, Hingorani AD, Hofman A, Holt PG, Hui J, Hunter ML, Imboden M, Jameson KA, Kerr SM, Kolcic I, Kronenberg F, Liu JZ, Marchini J, McKeever T, Morris AD, Olin AC, Porteous DJ, Postma DS, Rich SS, Ring SM, Rivadeneira F, Rochat T, Sayer AA, Sayers I, Sly PD, Smith GD, Sood A, Starr JM, Uitterlinden AG, Vonk JM, Wannamethee SG, Whincup PH, Wijmenga C, Williams OD, Wong A, Mangino M, Marciante KD, McArdle WL, Meibohm B, Morrison AC, North KE, Omenaas E, Palmer LJ, Pietilainen KH, Pin I, Pola Sbreve Ek O, Pouta A, Psaty BM, Hartikainen AL, Rantanen T, Ripatti S, Rotter JI, Rudan I, Rudnicka AR, Schulz H, Shin SY, Spector TD, Surakka I, Vitart V, Volzke H, Wareham NJ, Warrington NM, Wichmann HE, Wild SH, Wilk JB, Wjst M, Wright AF, Zgaga L, Zemunik T, Pennell CE, Nyberg F, Kuh D, Holloway JW, Boezen HM, Lawlor DA, Morris RW, Probst-Hensch N, Kaprio J, Wilson JF, Hayward C, Kahonen M, Heinrich J, Musk AW, Jarvis DL, Glaser S, Jarvelin MR, Ch Stricker BH, Elliott P, O'Connor GT, Strachan DP, London SJ, Hall IP, Gudnason V, Tobin MD. Genome-wide association and large-scale follow up identifies 16 new loci influencing lung function. *Nat Genet* 2011; 43: 1082-1090.

12. Miller S, Melen E, Merid SK, Hall IP, Sayers I. Genes associated with polymorphic variants predicting lung function are differentially expressed during human lung development. *Respir Res* 2016; 17: 95.

13. Howes A, Rogerson C, Belyaev N, Karagyozova T, Rapiteanu R, Fradique R, Pellicciotta N, Mayhew D, Hurd C, Crotta S, Singh T, Dingwell K, Myatt A, Arad N, Hasan H, Bijlsma H, Panjwani A, Vijayan V, Young G, Bridges A, Petit-Frere S, Betts J, Larminie C, Smith JC, Hessel EM, Michalovich D, Walport L, Cicuta P, Powell AJ, Beinke S, Wack A. The FAM13A Long Isoform Regulates Cilia Movement and Co-ordination in Airway Mucociliary Transport. *American Journal of Respiratory Cell and Molecular Biology* 2024; 0: null.

14. Jiang Z, Lao T, Qiu W, Polverino F, Gupta K, Guo F, Mancini JD, Naing ZZ, Cho MH, Castaldi PJ, Sun Y, Yu J, Laucho-Contreras ME, Kobzik L, Raby BA, Choi AM, Perrella MA, Owen CA, Silverman EK, Zhou X. A Chronic Obstructive Pulmonary Disease Susceptibility Gene, FAM13A, Regulates Protein Stability of beta-Catenin. *Am J Respir Crit Care Med* 2016; 194: 185-197.

15. Gong L, Bates S, Li J, Qiao D, Glass K, Wei W, Hsu VW, Zhou X, Silverman EK. Connecting COPD GWAS genes: FAM13A controls TGFβ2 secretion by modulating AP-3 transport. *Am J Respir Cell Mol Biol* 2021.

16. Zhu J, Wang F, Feng X, Li B, Ma L, Zhang J. Family with sequence similarity 13 member A mediates TGF-β1-induced EMT in small airway epithelium of patients with chronic obstructive pulmonary disease. *Respir Res* 2021; 22: 192.

17. Tam A, Leclair P, Li LV, Yang CX, Li X, Witzigmann D, Kulkarni JA, Hackett TL, Dorscheid DR, Singhera GK, Hogg JC, Cullis PR, Sin DD, Lim CJ. FAM13A as potential therapeutic target in modulating TGF-beta-induced airway tissue remodeling in COPD. *Am J Physiol Lung Cell Mol Physiol* 2021.

18. Jiang Z, Knudsen NH, Wang G, Qiu W, Naing ZZC, Bai Y, Ai X, Lee CH, Zhou X. Genetic Control of Fatty Acid beta-Oxidation in Chronic Obstructive Pulmonary Disease. *Am J Respir Cell Mol Biol* 2017; 56: 738-748.

19. Jin Z, Chung JW, Mei W, Strack S, He C, Lau GW, Yang J. Regulation of nuclear-cytoplasmic shuttling and function of Family with sequence similarity 13, member A (Fam13a), by B56-containing PP2As and Akt. *Mol Biol Cell* 2015; 26: 1160-1173.

20. Lin X, Li Y, Gong L, Yun JH, Xu S, Tesfaigzi Y, Qiao D, Zhou X. Tempo-spatial regulation of the Wnt pathway by FAM13A modulates the stemness of alveolar epithelial progenitors. *eBioMedicine* 2021; 69.

21. Goss AM, Tian Y, Tsukiyama T, Cohen ED, Zhou D, Lu MM, Yamaguchi TP, Morrisey EE. Wnt2/2b and β-Catenin Signaling Are Necessary and Sufficient to Specify Lung Progenitors in the Foregut. 2009; 17: 290-298.

22. Harris-Johnson KS, Domyan ET, Vezina CM, Sun X. β-Catenin promotes respiratory progenitor identity in mouse foregut. *Proceedings of the National Academy of Sciences* 2009; 106: 16287-16292.

23. Mucenski ML, Wert SE, Nation JM, Loudy DE, Huelsken J, Birchmeier W, Morrisey EE, Whitsett JA. beta-Catenin is required for specification of proximal/distal cell fate during lung morphogenesis. *J Biol Chem* 2003; 278: 40231-40238.

24. Shu W, Guttentag S, Wang Z, Andl T, Ballard P, Lu MM, Piccolo S, Birchmeier W, Whitsett JA, Millar SE, Morrisey EE. Wnt/beta-catenin signaling acts upstream of N-myc, BMP4, and FGF signaling to regulate proximal-distal patterning in the lung. *Dev Biol* 2005; 283: 226-239.

25. Okubo T, Hogan BL. Hyperactive Wnt signaling changes the developmental potential of embryonic lung endoderm. *J Biol* 2004; 3: 11.

26. Vazquez-Armendariz AI, Tata PR. Recent advances in lung organoid development and applications in disease modeling. *The Journal of Clinical Investigation* 2023; 133.

27. Green MD, Chen A, Nostro M-C, d'Souza SL, Schaniel C, Lemischka IR, Gouon-Evans V, Keller G, Snoeck H-W. Generation of anterior foregut endoderm from human embryonic and induced pluripotent stem cells. *Nature biotechnology* 2011; 29: 267.

28. Kubo A, Shinozaki K, Shannon JM, Kouskoff V, Kennedy M, Woo S, Fehling HJ, Keller G. Development of definitive endoderm from embryonic stem cells in culture. *Development (Cambridge, England)* 2004; 131: 1651-1662.

29. Hawkins F, Kramer P, Jacob A, Driver I, Thomas DC, McCauley KB, Skvir N, Crane AM, Kurmann AA, Hollenberg AN, Nguyen S, Wong BG, Khalil AS, Huang SX, Guttentag S, Rock JR, Shannon JM, Davis BR, Kotton DN. Prospective isolation of NKX2-1-expressing human lung progenitors derived from pluripotent stem cells. *J Clin Invest* 2017; 127: 2277-2294.

30. Longmire TA, Ikonomou L, Hawkins F, Christodoulou C, Cao Y, Jean JC, Kwok LW, Mou H, Rajagopal J, Shen SS, Dowton AA, Serra M, Weiss DJ, Green MD, Snoeck HW, Ramirez MI, Kotton DN. Efficient derivation of purified lung and thyroid progenitors from embryonic stem cells. *Cell stem cell* 2012; 10: 398-411.

31. Hawkins FJ, Suzuki S, Beermann ML, Barillà C, Wang R, Villacorta-Martin C, Berical A, Jean JC, Le Suer J, Matte T, Simone-Roach C, Tang Y, Schlaeger TM, Crane AM, Matthias N, Huang SXL, Randell SH, Wu J, Spence JR, Carraro G, Stripp BR, Rab A, Sorsher EJ, Horani A, Brody SL, Davis BR, Kotton DN. Derivation of Airway Basal Stem Cells from Human Pluripotent Stem Cells. *Cell stem cell* 2021; 28: 79-95.e78.

32. Jacob A, Morley M, Hawkins F, McCauley KB, Jean JC, Heins H, Na CL, Weaver TE, Vedaie M, Hurley K, Hinds A, Russo SJ, Kook S, Zacharias W, Ochs M, Traber K, Quinton LJ, Crane A, Davis BR, White FV, Wambach J, Whitsett JA, Cole FS, Morrisey EE, Guttentag SH, Beers MF, Kotton DN. Differentiation of Human Pluripotent Stem Cells into Functional Lung Alveolar Epithelial Cells. *Cell stem cell* 2017; 21: 472-488 e410.

33. McCauley KB, Hawkins F, Serra M, Thomas DC, Jacob A, Kotton DN. Efficient Derivation of Functional Human Airway Epithelium from Pluripotent Stem Cells via Temporal Regulation of Wnt Signaling. *Cell stem cell* 2017; 20: 844-857 e846.

34. Burgess CL, Huang J, Bawa PS, Alysandratos K-D, Minakin K, Ayers LJ, Morley MP, Babu A, Villacorta-Martin C, Yampolskaya M, Hinds A, Thapa BR, Wang F, Matschulat A, Mehta P, Morrisey EE, Varelas X, Kotton DN. Generation of human alveolar epithelial type I cells from pluripotent stem cells. *Cell stem cell* 2024.

35. Werder RB, Huang J, Abo KM, Hix OT, Minakin K, Alysandratos K-D, Merritt C, Berthiaume K, Alber AB, Burgess CL, Kotton DN, Wilson AA. Generating 3D Spheres and 2D Air-Liquid Interface Cultures of Human Induced Pluripotent Stem Cell-Derived Type 2 Alveolar Epithelial Cells. *Journal of Visualized Experiments* 2022: e63875.

36. Jacob A, Vedaie M, Roberts DA, Thomas DC, Villacorta-Martin C, Alysandratos KD, Hawkins F, Kotton DN. Derivation of self-renewing lung alveolar epithelial type II cells from human pluripotent stem cells. *Nature protocols* 2019; 14: 3303-3332.

37. Werder RB, Kaserman JE, Packer MS, Lindstrom-Vautrin J, Villacorta-Martin C, Young LE, Aratyn-Schaus Y, Gregoire F, Wilson AA. Adenine base editing reduces misfolded protein accumulation and toxicity in alpha-1 antitrypsin deficient patient iPSC-hepatocytes. *Molecular therapy : the journal of the American Society of Gene Therapy* 2021; 29: 3219-3229.

38. Wilson AA, Ying L, Liesa M, Segeritz CP, Mills JA, Shen SS, Jean J, Lonza GC, Liberti DC, Lang AH, Nazaire J, Gower AC, Mueller FJ, Mehta P, Ordonez A, Lomas DA, Vallier L, Murphy GJ, Mostoslavsky G, Spira A, Shirihai OS, Ramirez MI, Gadue P, Kotton DN. Emergence of a stage-dependent human liver disease signature with directed differentiation of alpha-1 antitrypsin-deficient iPS cells. *Stem cell reports* 2015; 4: 873-885.

39. Li B, Dewey CN. RSEM: accurate transcript quantification from RNA-Seq data with or without a reference genome. *BMC Bioinformatics* 2011; 12: 323.

40. Veeman MT, Slusarski DC, Kaykas A, Louie SH, Moon RT. Zebrafish prickle, a modulator of noncanonical Wnt/Fz signaling, regulates gastrulation movements. *Curr Biol* 2003; 13: 680-685.

41. Ten Berge D, Koole W, Fuerer C, Fish M, Eroglu E, Nusse R. Wnt Signaling Mediates Self-Organization and Axis Formation in Embryoid Bodies. *Cell stem cell* 2008; 3: 508-518.

42. Baloban M, Shcherbakova DM, Pletnev S, Pletnev VZ, Lagarias JC, Verkhusha VV. Designing brighter near-infrared fluorescent proteins: insights from structural and biochemical studies. *Chem Sci* 2017; 8: 4546-4557.

43. Mostoslavsky G, Fabian AJ, Rooney S, Alt FW, Mulligan RC. Complete correction of murine Artemis immunodeficiency by lentiviral vector-mediated gene transfer. *Proc Natl Acad Sci U S A* 2006; 103: 16406-16411.

44. Wilson AA, Kwok LW, Porter EL, Payne JG, McElroy GS, Ohle SJ, Greenhill SR, Blahna MT, Yamamoto K, Jean JC, Mizgerd JP, Kotton DN. Lentiviral delivery of RNAi for in vivo lineage-specific modulation of gene expression in mouse lung macrophages. *Molecular therapy : the journal of the American Society of Gene Therapy* 2013; 21: 825-833.

45. Federico A, Monti S. hypeR: an R package for geneset enrichment workflows. *Bioinformatics (Oxford, England)* 2020; 36: 1307-1308.

46. Garcia-Alonso L, Holland CH, Ibrahim MM, Turei D, Saez-Rodriguez J. Benchmark and integration of resources for the estimation of human transcription factor activities. *Genome research* 2019; 29: 1363-1375.

47. Werder RB, Zhou X, Cho MH, Wilson AA. Breathing new life into the study of COPD with genes identified from genome-wide association studies. *European Respiratory Review* 2024; 33: 240019.

48. Serra M, Alysandratos KD, Hawkins F, McCauley KB, Jacob A, Choi J, Caballero IS, Vedaie M, Kurmann AA, Ikonomou L, Hollenberg AN, Shannon JM, Kotton DN. Pluripotent stem cell differentiation reveals distinct developmental pathways regulating lung- versus thyroid-lineage specification. *Development (Cambridge, England)* 2017; 144: 3879-3893.

49. Werder RB, Liu T, Abo KM, Lindstrom-Vautrin J, Villacorta-Martin C, Huang J, Hinds A, Boyer N, Bullitt E, Liesa M, Silverman EK, Kotton DN, Cho MH, Zhou X, Wilson AA. CRISPR interference interrogation of COPD GWAS genes reveals the functional significance of desmoplakin in iPSC-derived alveolar epithelial cells. *Science Advances* 2022; 8: eabo6566.

50. Lazzaro D, Price M, de Felice M, Di Lauro R. The transcription factor TTF-1 is expressed at the onset of thyroid and lung morphogenesis and in restricted regions of the foetal brain. *Development (Cambridge, England)* 1991; 113: 1093-1104.

51. Mithal A, Capilla A, Heinze D, Berical A, Villacorta-Martin C, Vedaie M, Jacob A, Abo K, Szymaniak A, Peasley M, Stuffer A, Mahoney J, Kotton DN, Hawkins F, Mostoslavsky G. Generation of mesenchyme free intestinal organoids from human induced pluripotent stem cells. *Nature Communications* 2020; 11: 215.

52. Danopoulos S, Alonso I, Thornton ME, Grubbs BH, Bellusci S, Warburton D, Al Alam D. Human lung branching morphogenesis is orchestrated by the spatiotemporal distribution of ACTA2, SOX2, and SOX9. *Am J Physiol Lung Cell Mol Physiol* 2018; 314: L144-l149.

53. Danopoulos S, Bellusci S, Warburton D, Al Alam D. Identification of a SOX2/SOX9 Double Positive Cells Progenitor Cell Population Required for Branching Morphogenesis in Human Lung. *The FASEB Journal* 2017; 31: 872.871-872.871.

54. Minoo P, Hu L, Xing Y, Zhu NL, Chen H, Li M, Borok Z, Li C. Physical and functional interactions between homeodomain NKX2.1 and winged helix/forkhead FOXA1 in lung epithelial cells. *Mol Cell Biol* 2007; 27: 2155-2165.

55. Chen B, Dodge ME, Tang W, Lu J, Ma Z, Fan CW, Wei S, Hao W, Kilgore J, Williams NS, Roth MG, Amatruda JF, Chen C, Lum L. Small molecule-mediated disruption of Wnt-dependent signaling in tissue regeneration and cancer. *Nat Chem Biol* 2009; 5: 100-107.

56. Ruffin M, Thompson KE, Corvol H, Guillot L. Two-hybrid screening of FAM13A protein partners in lung epithelial cells. *BMC Res Notes* 2020; 12: 804.

57. Li Y, Rankin SA, Sinner D, Kenny AP, Krieg PA, Zorn AM. Sfrp5 coordinates foregut specification and morphogenesis by antagonizing both canonical and noncanonical Wnt11 signaling. *Genes & development* 2008; 22: 3050-3063.

58. McLin VA, Rankin SA, Zorn AM. Repression of Wnt/beta-catenin signaling in the anterior endoderm is essential for liver and pancreas development. *Development (Cambridge, England)* 2007; 134: 2207-2217.

59. Funa NS, Mjoseng HK, de Lichtenberg KH, Raineri S, Esen D, Egeskov-Madsen AlR, Quaranta R, Jørgensen MC, Hansen MS, van Cuyl Kuylenstierna J, Jensen KB, Miao Y, Garcia KC, Seymour PA, Serup P. TGF-B modulates cell fate in human ES cell-derived foregut endoderm by inhibiting Wnt and BMP signaling. *Stem cell reports* 2024; 19: 973-992.

60. Nattes E, Lejeune S, Carsin A, Borie R, Gibertini I, Balinotti J, Nathan N, Marchand-Adam S, Thumerelle C, Fauroux B, Bosdure E, Houdouin V, Delestrain C, Louha M, Couderc R, De Becdelievre A, Fanen P, Funalot B, Crestani B, Deschildre A, Dubus JC, Epaud R. Heterogeneity of lung disease associated with NK2 homeobox 1 mutations. *Respir Med* 2017; 129: 16-23.

61. Hamvas A, Deterding RR, Wert SE, White FV, Dishop MK, Alfano DN, Halbower AC, Planer B, Stephan MJ, Uchida DA, Williames LD, Rosenfeld JA, Lebel RR, Young LR, Cole FS, Nogee LM. Heterogeneous pulmonary phenotypes associated with mutations in the thyroid transcription factor gene NKX2-1. *Chest* 2013; 144: 794-804.

62. Delestrain C, Aissat A, Nattes E, Gibertini I, Lacroze V, Simon S, Decrouy X, de Becdelièvre A, Fanen P, Epaud R. Deciphering an isolated lung phenotype of NKX2-1 frameshift pathogenic variant. *Front Pediatr* 2022; 10: 978598.

63. Maquet E, Costagliola S, Parma J, Christophe-Hobertus C, Oligny LL, Fournet JC, Robitaille Y, Vuissoz JM, Payot A, Laberge S, Vassart G, Van Vliet G, Deladoëy J. Lethal respiratory failure and mild primary hypothyroidism in a term girl with a de novo heterozygous mutation in the TITF1/NKX2.1 gene. *J Clin Endocrinol Metab* 2009; 94: 197-203.

64. Minoo P, Su G, Drum H, Bringas P, Kimura S. Defects in Tracheoesophageal and Lung Morphogenesis inNkx2.1(−/−) Mouse Embryos. *Developmental Biology* 1999; 209: 60-71.

65. Hawkins F, Rankin SA, Kotton DN, Zorn AM. The Genetic Programs Regulating Embryonic Lung Development and Induced Pluripotent Stem Cell Differentiation. 2015.

66. Alysandratos KD, Russo SJ, Petcherski A, Taddeo EP, Acin-Perez R, Villacorta-Martin C, Jean JC, Mulugeta S, Rodriguez LR, Blum BC, Hekman RM, Hix OT, Minakin K, Vedaie M, Kook S, Tilston-Lunel AM, Varelas X, Wambach JA, Cole FS, Hamvas A, Young LR, Liesa M, Emili A, Guttentag SH, Shirihai OS, Beers MF, Kotton DN. Patient-specific iPSCs carrying an SFTPC mutation reveal the intrinsic alveolar epithelial dysfunction at the inception of interstitial lung disease. *Cell reports* 2021; 36: 109636.

67. Sun YL, Hennessey EE, Heins H, Yang P, Villacorta-Martin C, Kwan J, Gopalan K, James M, Emili A, Cole FS, Wambach JA, Kotton DN. Human pluripotent stem cell modeling of alveolar type 2 cell dysfunction caused by ABCA3 mutations. *J Clin Invest* 2024; 134.

68. Travaglini KJ, Nabhan AN, Penland L, Sinha R, Gillich A, Sit RV, Chang S, Conley SD, Mori Y, Seita J, Berry GJ, Shrager JB, Metzger RJ, Kuo CS, Neff N, Weissman IL, Quake SR, Krasnow MA. A molecular cell atlas of the human lung from single-cell RNA sequencing. *Nature* 2020; 587: 619-625.

**Supplemental Figure 1.** Related to Figure 1. A) Expression of long or short *FAM13A* isoforms using ENSEMBL IDs in iPSCs, iPSC-derived lung progenitors (D15) or iPSC-derived type 2 alveolar epithelial cells (D35), or in primary fetal or adult type 2 alveolar epithelial cells (29, 32). B) Normal karyotype of FAM13A long isoform heterozygous (left) and knockout (right) mutant iPSCs.

**Supplemental Figure 2.** Related to Figure 2-3. A) Expression of *MKI67* following two weeks of lung progenitors in proximal or distal media, quantified by qRT-PCR. B) FAM13A long isoform wildtype (WT), heterozygous (HET) or knockout (KO) mutant iPSCs were tested for their ability to derive germ layers, or mature non-lung endoderm lineages. Endoderm induction efficiency was quantified by CXCR4+ cKit+ cells by flow cytometry. C) Mesoderm induction quantified by Brachyury+ NCAM+ cells by flow cytometry. D) Ectoderm induction quantified by PAX6+ Nestin+ cells by flow cytometry. E) Efficiency of iPSC-derived hepatocytes differentiation quantified by AFP+ FOXA1+ cells by flow cytometry. n=3-6 experimental replicates; error bars represent SD. Statistical significance was determined by one-way ANOVA with a Tukey multiple comparison test; ****p < 0.0001.

**Supplemental Figure 3.** Related to Figure 3. FAM13A long isoform wildtype (WT, green), heterozygous (HET, orange) or knockout (KO, purple) mutant iPSCs differentiated to lung progenitors expressing NKX2-1 (GFP+) or surrounding non-lung endoderm (GFP-). A) Expression of the FAM13A-long isoform in GFP- or GFP+ sorted cell populations, measured by qRT-PCR. B) Expression of the thyroid marker *PAX8* or C) brain marker *OTX2* measured by qRT-PCR. D) Quantification of CDX2+ cells from immunostaining of D15 cultures. E) Expression of liver (*AFP*), F) stomach (*TFF1*), G) intestine (*CDX2*), H) esophagus (*TP63*) or I) pancreas (*PDX1*) in GFP- or GFP+ sorted cell populations, measured by qRT-PCR, relative to an average of D0 samples. J) Expression of pan-endoderm marker, *FOXA2*, in GFP- or GFP+ sorted cell populations, measured by qRT-PCR. n=3 experimental replicates; error bars represent SD. Statistical significance was determined by one-way ANOVA with a Tukey multiple comparison test; **p < 0.005, ***p < 0.001.

**Supplemental Figure 4.** Related to Figure 4. A) Feature plot of *NKX2-1, CDX2* and *HHEX* expression of uniform manifold projection (UMAP) in Figure 4B. B) Cell cycle phase in scRNA-seq of D15 NKX2-1+ cells in Figure 4C. C) Dot plot showing expression of lung progenitor and endoderm genes (29) in FAM13A long isoform WT or KO D15 NKX2-1+ cells. D) Top 10 pathways identified by gene-set enrichment analysis of FAM13A long isoform WT or KO D15 NKX2-1+ cells. E) Regulon analysis of FAM13A long isoform WT or KO D15 NKX2-1+ cells.

**Supplemental Figure 5.** Related to Figure 4. A) scRNA-seq analysis of FAM13A long isoform WT or KO D15 NKX2-1- cells. B) Uniform manifold projection (UMAP) of FAM13A long isoform WT or KO D15 NKX2-1- cells, showing original identity (top) or cell cycle phase (bottom). C) Louvain clustering at resolutions from 0.1-1. D) Heatmap showing differentially expressed genes between FAM13A long isoform WT (blue) and KO (pink) D15 NKX2-1- cells. E) Normalized expression of non-lung endoderm genes in D15 NKX2-1- cells. F) Module score of iPSC-derived gut markers (45). G) Gene-set enrichment analysis of FAM13A long isoform WT or KO D15 NKX2-1- cells.

**Supplemental Figure 6.** Related to Figure 4. A) scRNA-seq analysis of FAM13A long isoform WT or KO D6 anterior foregut endoderm (AFE) cells. Uniform manifold projection (UMAP) of FAM13A long isoform WT or KO AFE cells, showing original identity. B) UMAP of Louvain clustering at resolutions from 0.1-1. C) UMAP of cell cycle phase. D) Heatmap showing differentially expressed genes between FAM13A long isoform WT (gold) and KO (turquoise) D6 AFE cells. E) Module score of anterior foregut endoderm genes in FAM13A long isoform WT and KO D6 AFE cells (29). F) Module score of lung progenitor genes in FAM13A long isoform WT and KO D6 AFE cells (29). G) Normalized expression of anterior and posterior foregut genes in FAM13A long isoform WT and KO D6 AFE cells. H) Normalized expression of differentially expressed Wnt activation (*WLS*) and Wnt target (*SP5, CCND1*) genes in FAM13A long isoform WT and KO D6 AFE cells.

**Supplemental Table 1.** Summary of normality testing and statistical tests applied to each dataset throughout the manuscript.
