# Supplementary material for: Long isoforms of the COPD risk gene FAM13A orchestrate human lung epithelial development
Source: Am J Respir Cell Mol Biol. Author manuscript; Available in PMC 2026 Jul 6. (PMC13336431; doi:10.1093/ajrcmb/aanag078)

Supplementary Figure 1

A

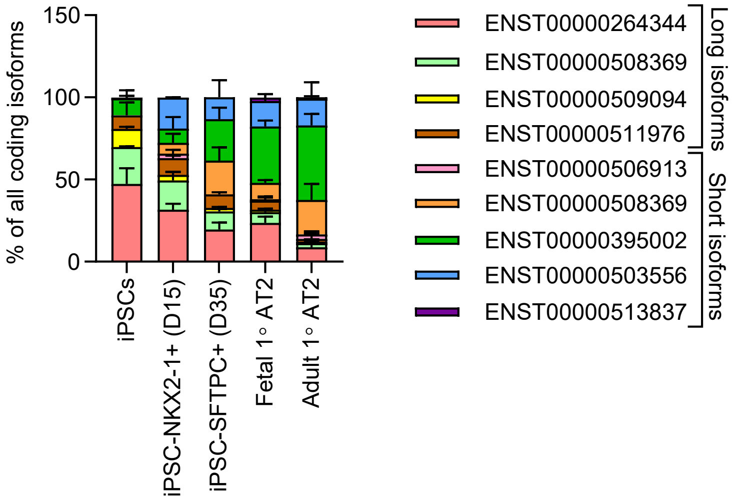

B

FAM13A long isoform heterozygous mutant

Result: 46,XY

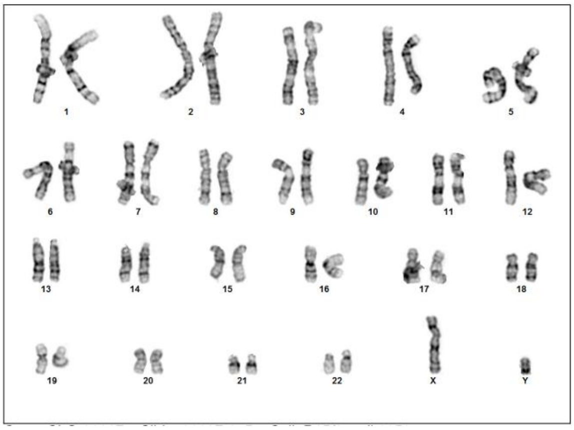

FAM13A long isoform homozygous mutant

Result: 46,XY

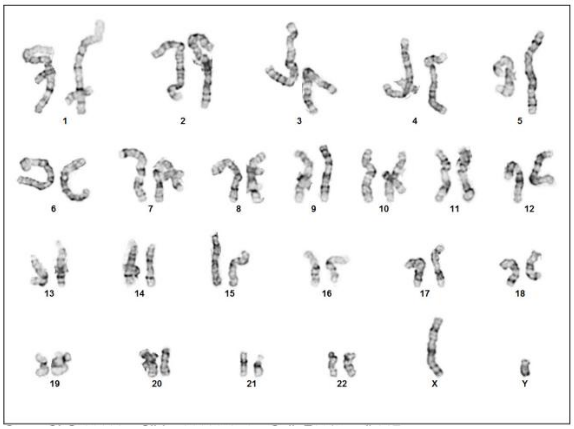

Supplementary Figure 2

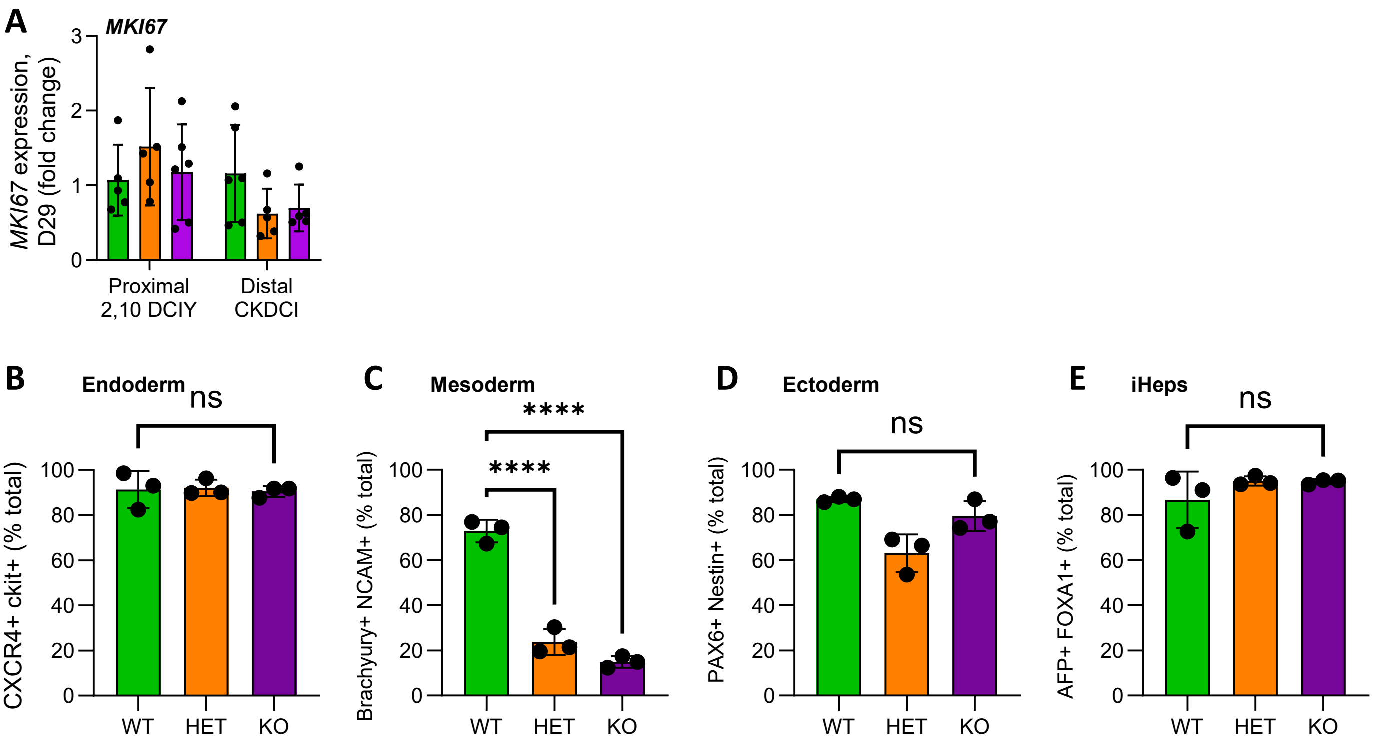

Supplemental Figure 3

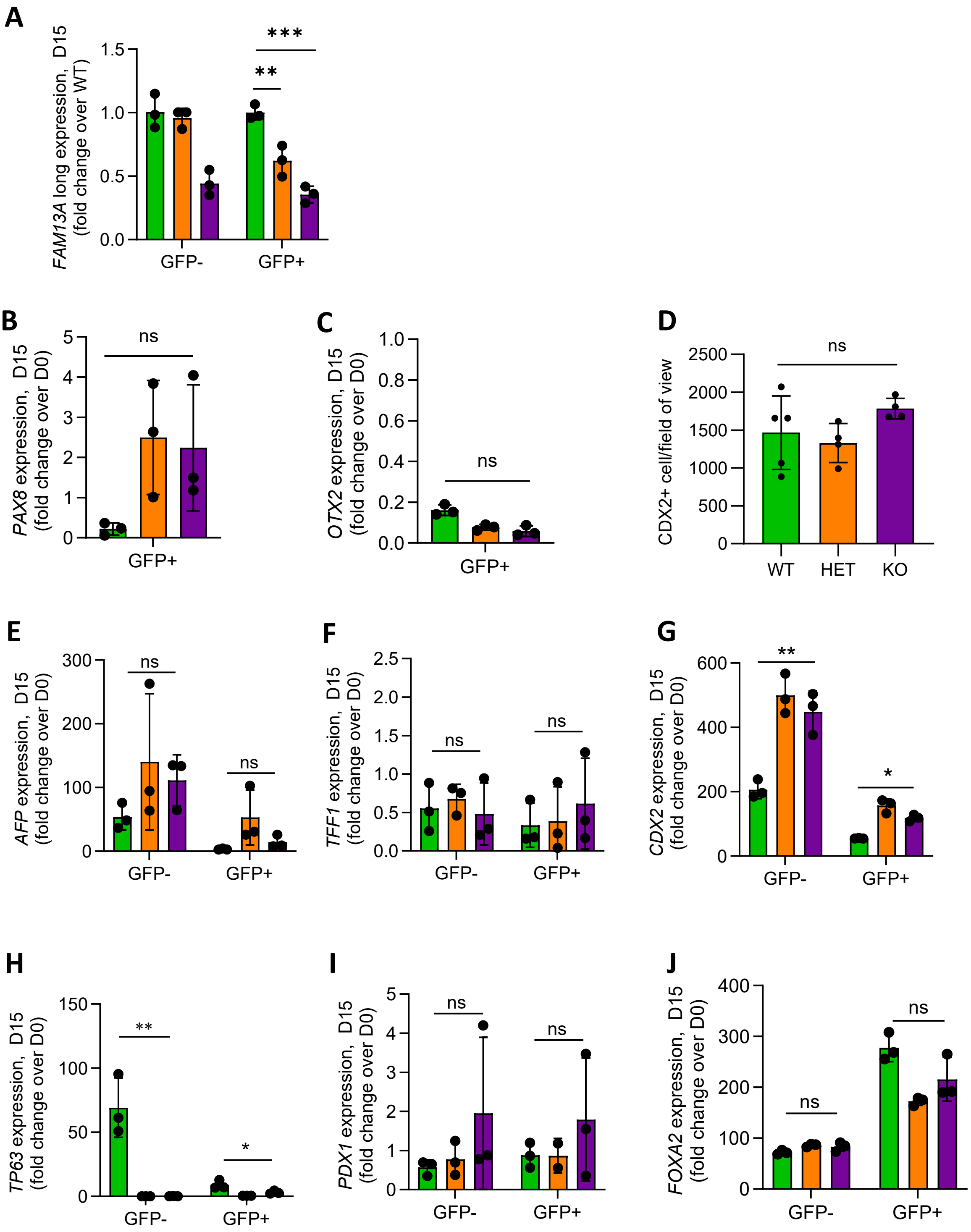

Supplemental Figure 4

A

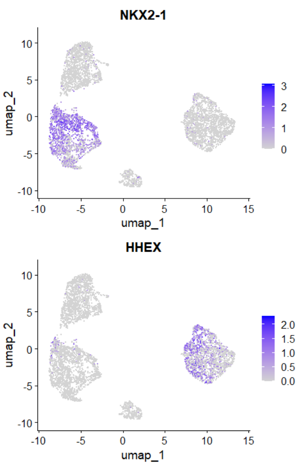

B

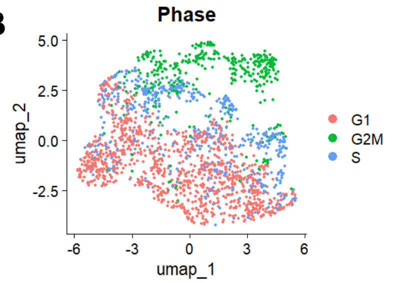

C

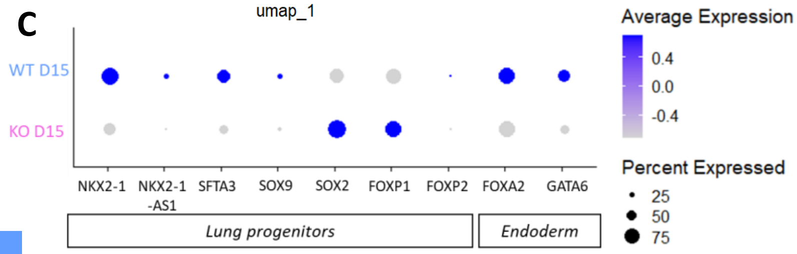

D

Upregulated in WT NKX2-1+ D15	pval	fdr
GO_EPITHELIUM_DEVELOPMENT	8.80E-12	6.60E-08
GO_REPRODUCTIVE_SYSTEM_DEVELOPMENT	7.30E-11	2.80E-07
GO_RESPIRATORY_SYSTEM_DEVELOPMENT	1.40E-10	3.60E-07
GO_TUBE_DEVELOPMENT	2.10E-10	3.90E-07
GO_UROGENITAL_SYSTEM_DEVELOPMENT	5.50E-09	8.40E-06
GO_MORPHOGENESIS_OF_AN_EPITHELIUM	1.50E-08	1.90E-05
GO_METANEPHROS_DEVELOPMENT	4.60E-08	4.50E-05
GO_BIOLOGICAL_ADHESION	4.80E-08	4.50E-05
GO_REPRODUCTION	6.40E-08	5.40E-05
GO_TUBE_MORPHOGENESIS	8.10E-08	6.10E-05

Upregulated in KO NKX2-1+ D15	pval	fdr
GO_RESPONSE_TO_ENDOGENOUS_STIMULUS	3.40E-07	0.0019
GO_RESPONSE_TO_STEROID_HORMONE	5.00E-07	0.0019
GO_NAD_METABOLIC_PROCESS	1.00E-06	0.0026
GO_RESPONSE_TO_ORGANIC_CYCLIC_COMPOUND	1.40E-06	0.0026
GO_RESPONSE_TO_CORTICOSTEROID	2.20E-06	0.0029
GO_TUBE_DEVELOPMENT	3.10E-06	0.0029
GO_GLYCOLYTIC_PROCESS_THROUGH_FRUCTOSE_6_PHOSPHATE	3.60E-06	0.0029
GO_REGULATION_OF_CELL_POPULATION_PROLIFERATION	3.70E-06	0.0029
GO_POSITIVE_REGULATION_OF_DEVELOPMENTAL_PROCESS	4.10E-06	0.0029
GO_RESPONSE_TO_HORMONE	4.30E-06	0.0029
GO_GLAND_DEVELOPMENT	4.60E-06	0.0029

E

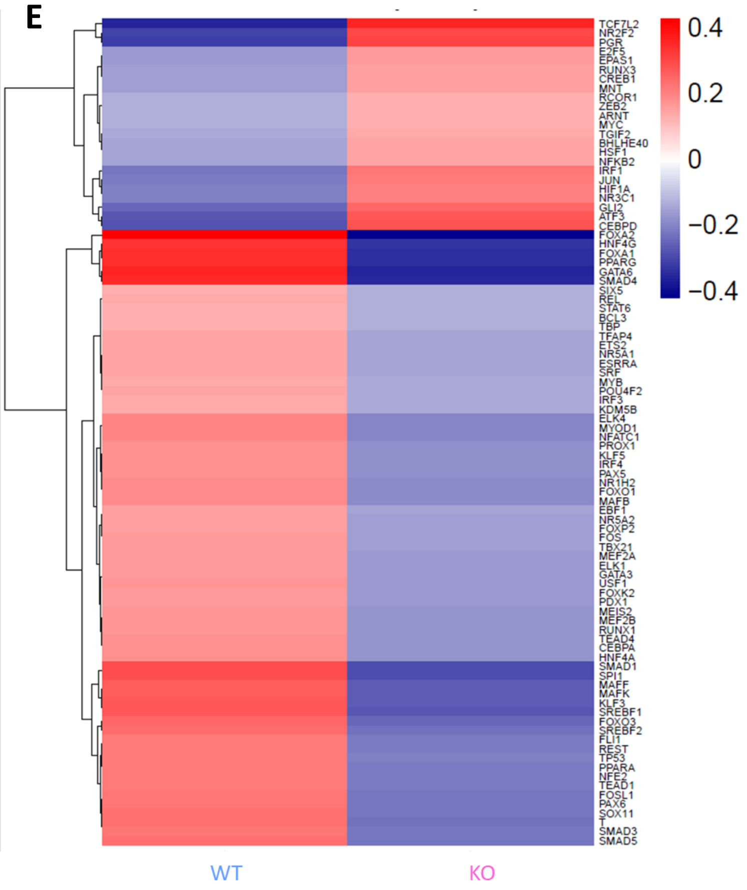

Supplemental Figure 5

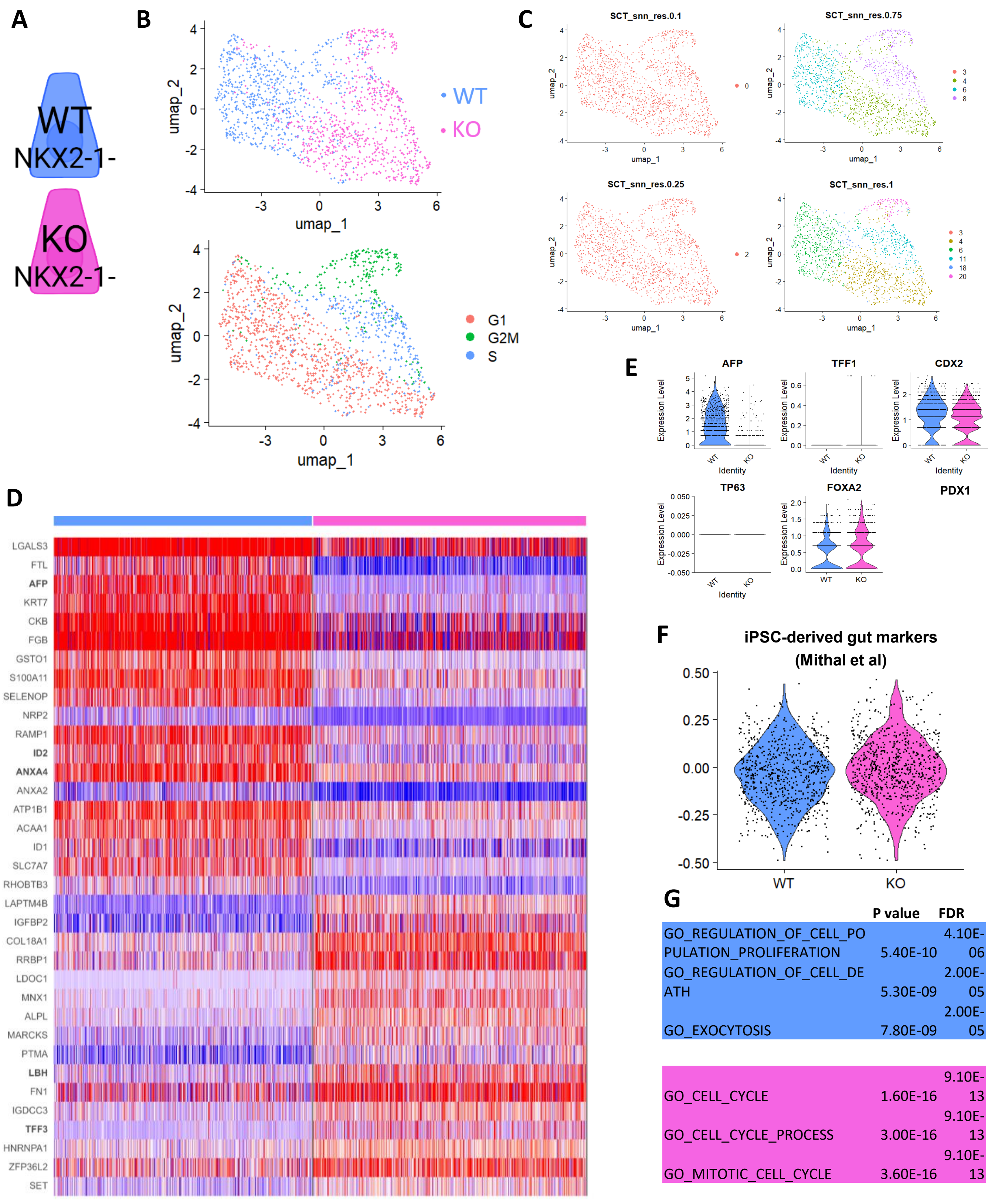

	P value	FDR
GO_REGULATION_OF_CELL_POPULATION_PROLIFERATION	5.40E-10	4.10E-06
GO_REGULATION_OF_CELL_DEATH	5.30E-09	2.00E-05
GO_EXOCYTOSIS	7.80E-09	2.00E-05
GO_CELL_CYCLE	1.60E-16	9.10E-13
GO_CELL_CYCLE_PROCESS	3.00E-16	9.10E-13
GO_MITOTIC_CELL_CYCLE	3.60E-16	9.10E-13

Supplemental Figure 6

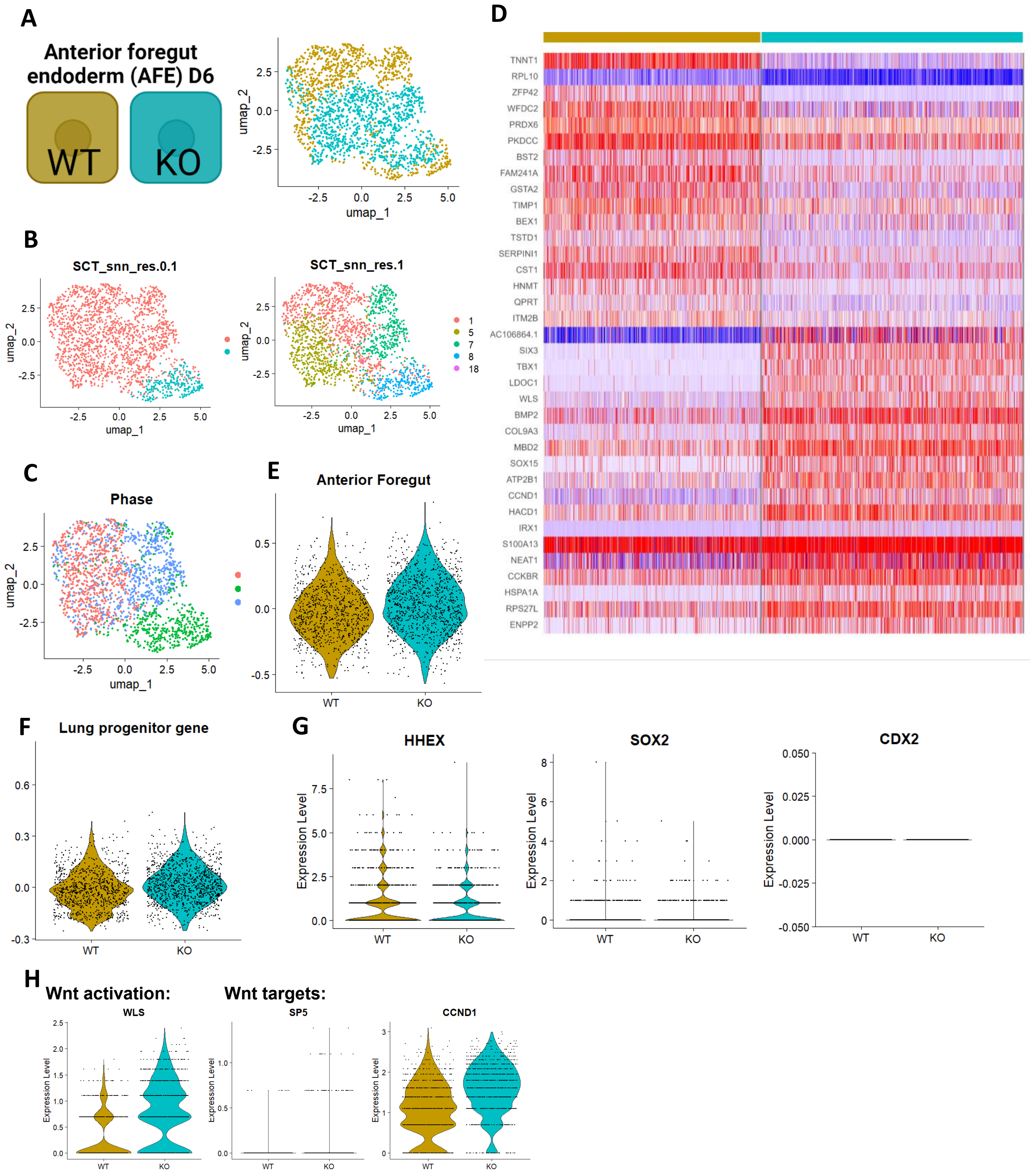

Supplement: Supplementary Figures [file NIHMS2185259-supplement-Supplementary_Figures.pdf]
